# Effective interventions to ensure MCH (Maternal and Child Health) services during pandemic related health emergencies (Zika, Ebola, and COVID-19): A systematic review

**DOI:** 10.1371/journal.pone.0268106

**Published:** 2022-05-10

**Authors:** Subrata Kumar Palo, Shubhankar Dubey, Sapna Negi, Mili Roopchand Sahay, Kripalini Patel, Swagatika Swain, Bijaya Kumar Mishra, Dinesh Bhuyan, Srikanta Kanungo, Meena Som, Brajesh Raj Merta, Debdutta Bhattacharya, Jaya Singh Kshatri, Sanghamitra Pati

**Affiliations:** 1 ICMR-Regional Medical Research Centre, Bhubaneswar, Odisha, India; 2 United Nations Children’s Fund (UNICEF), Odisha, India; Jatiya Kabi Kazi Nazrul Islam University, BANGLADESH

## Abstract

**Introduction:**

Ensuring accessible and quality health care for women and children is an existing challenge, which is further exacerbated during pandemics. There is a knowledge gap about the effect of pandemics on maternal, newborn, and child well-being. This systematic review was conducted to study maternal and child health (MCH) services utilization during pandemics (Zika, Ebola, and COVID-19) and the effectiveness of various interventions undertaken for ensuring utilization of MCH services.

**Methodology:**

A systematic and comprehensive search was conducted in MEDLINE/PubMed, Cochrane CENTRAL, Embase, Epistemonikos, ScienceDirect, and Google Scholar. Of 5643 citations, 60 potential studies were finally included for analysis. The included studies were appraised using JBI Critical appraisal tools. Study selection and data extraction were done independently and in duplicate. Findings are presented narratively based on the RMNCHA framework by World Health Organization (WHO).

**Results:**

Maternal and child health services such as antenatal care (ANC) visits, institutional deliveries, immunization uptake, were greatly affected during a pandemic situation. Innovative approaches in form of health care services through virtual consultation, patient triaging, developing dedicated COVID maternity centers and maternity schools were implemented in different places for ensuring continuity of MCH care during pandemics. None of the studies reported the effectiveness of these interventions during pandemic-related health emergencies.

**Conclusion:**

The findings suggest that during pandemics, MCH care utilization often gets affected. Many innovative interventions were adopted to ensure MCH services. However, they lack evidence about their effectiveness. It is critically important to implement evidence-based appropriate interventions for better MCH care utilization.

## Introduction

The health of women and children is critical for a happier and healthier world. They have been classified by the World Health Organization (WHO) as an important subgroup of the population that is most vulnerable in a pandemic or a disaster [1]. Globally, the COVID-19 pandemic has posed a challenge to the health systems leading to a compromise on health care services that embrace maternal and child health (MCH) care. Similar lapses were also witnessed during past health emergencies (Ebola and Zika), implying lessons are to be learned from the present and past epidemics or pandemics [2, 3].

In developing countries, ensuring accessible and quality health care to women and children has been an existing challenge, further exacerbated due to pandemics (Zika, Ebola, and COVID-19). Maternal and child health care is affected due to various factors that influence MCH care administration, service provision, and uptake of the services by the beneficiaries (pregnant women, mothers, and children) [4]. Previous health emergencies like Ebola and Zika have reported a significant increase in the maternal mortality ratio (MMR) due to decreased approach to health facilities and increased risky home deliveries [5]. The administrator’s focus will likely to shift from MCH care to the pandemic. According to research, health workers in other Sub-Saharan African countries are not well-prepared to treat COVID patients and meet the demands of women during the pandemic [6]. Research has revealed a knowledge gap regarding the effect of pandemics on maternal well-being, especially in resource-constrained settings where marginalized women often receive poor health care [7]. A study conducted in low- and middle-income countries (LMICs) has found a declining trend in the utilization of maternal and child health (MCH) services such as institutional delivery, antenatal care (ANC), and child immunization [8]. These limitations may have severe consequences for women’s health in LMICs during the pandemic [9].

Furthermore, many pregnant women have found it challenging to access healthcare facilities due to the lockdown and movement limits. Due to lack of transportation, pregnant women in Panama and Zimbabwe have reported trouble getting to a health facility [10, 11]. Owing to travel limits and far-flung pharmacies, pregnant women in Zimbabwe had problems finding treatment for their newborns [10]. Several Indian states have witnessed a decline in institutional deliveries [12]. There have been incidents of pregnant women giving birth on the road and in ambulances due to lockdown and delays in getting emergency services [13–15].

Although health systems around the globe have implemented extraordinary measures to prevent COVID-19 transmission, such measures have negatively impacted maternal and neonatal health and exacerbated the existing inequalities within societies [16]. Fighting with health emergencies and maintaining a continuum of care through routine essential services was challenging. The strict pandemic control policies on healthcare infrastructure, societies, and the global economy also affected maternal health [17]. The need of the situation is to maintain the “continuum of care” aiming at delivering the services to mothers and children through an integrated approach [18].

With this backdrop, the present systematic review was conducted to assess the impact of health emergencies (Zika, Ebola, and COVID-19) on facility and community-based MCH services utilization and identify various effective interventions or strategies adopted to ensure uptake/delivery of MCH care. This review will offer evidence to key stakeholders at different levels of the healthcare system for developing and implementing a straightforward approach with a context-specific strategic plan to overcome pandemic-related negative consequences on MCH care.

## Methodology

The PRISMA (Preferred Reporting Items for Systematic Reviews and Meta-Analyses) [19] guidelines were followed for this systematic review. The protocol for this systematic review was registered in PROSPERO with ID: CRD42021233860.

## Search

A comprehensive search was conducted in MEDLINE/PubMed, Cochrane CENTRAL, Embase, ProQuest, Google Scholar, Epistemonikos, and ScienceDirect using a predefined search strategy based on population, intervention/phenomenon of interest, comparator/context, and outcomes (**S1 Data**).

## Study selection

Based on predefined inclusion and exclusion criteria, reviewers (SD, SN, SS, and KP) screened the title and abstract of identified studies. Reviewers (SD, SN, SS, KP, and MRS) carried out the full-text screening of potentially relevant studies. The entire study selection was made independently and in duplicate.

### Criteria for inclusion/exclusion of studies

#### Study design and period

Any quantitative, qualitative, and mixed-method studies irrespective of settings were involved. Studies reporting on access and utilization of antenatal and postnatal services, intrapartum care, referral services, immunization services, and sick child care services were included. However, studies such as reviews, case reports, editorials, commentaries, perspectives, and articles with methodological flaws were excluded. Though grey literature regarding this topic was numerous, we restricted the search to peer-reviewed articles as it signifies that the quality of articles was checked before publication. No limit was applied to the language; non-English studies were handled using Google Translate. All studies published till January 2021 were included in this review.

#### Population/participants

Pregnant women, mothers, children, and health care professionals.

#### Intervention(s)/exposure(s)/phenomenon of interest

Any interventions or strategies related to providing or improving MCH services during health emergencies (Zika, Ebola, and COVID-19).

#### Comparator/context

There was no comparator group. Studies in the context of health emergencies (Zika, Ebola, and COVID-19) were included.

#### Outcome

WHO MCH care indicators in alignment with the RMNCH (Reproductive, Maternal, Newborn and Child Health) “Continuum of Care” framework [18, 20], such as demand for family planning, antenatal care coverage, institutional deliveries, maternal and perinatal outcomes, postnatal care within two days of birth, immunization services and pediatric health services during health emergencies (Zika, Ebola, and COVID-19) were studied.

Two reviewers (BKM, SKP) resolved the disagreement between authors through discussion to reach a consensus at each screening stage.

### Appraisal of studies

The methodological quality of the included studies was appraised using JBI tools (Joanna Briggs Institute) [21] by the authors (SD, SN, KP, SS, MRS) independently. The quality rating in the included studies that scored > 70% were considered high quality, whereas articles scoring between 40–70% and < 40% were considered moderate and low quality, respectively based on the scores for the individual items as decided with consensus.

Any disagreements were resolved in concordance with the reviewers’ SKP and BKM.

### Data extraction, synthesis, and analysis

Reviewers (SD, SN, KP, SS, and MRS) extracted data independently from the included studies and cross-checked it with other reviewers. Data were extracted for the following study characteristics viz. author/year, title, study year, objectives, study type, sample size, study design, study setting, country, participants, method of data collection, pandemic type (Zika, Ebola, and COVID-19), methods of analysis, outcomes (based on RMNCH indicators), results and conclusion.

PICO components of each study were summarized, characterized, and labeled into specific domains (Family Planning, Reproductive, Maternal, Newborn, and Child Health). Descriptive statistics were used to present quantitative data based on their characteristics and availability. The synthesis of the qualitative data was done using thematic analysis. The reviewers independently read and re-read the data line-by-line from the results of the included primary studies. The codes were grouped to generate the descriptive themes, which were further examined, compared, and refined to generate analytical themes. The data coding was done using identified themes with the help of MAXQDA 2020 (Version 20.4.1). The reviewers sorted the data by theme and presented the themes in the form of a table of analysis (**Table 1**).

**Table 1:** Qualitative finding analysis.

Maternal and child health Interventions	Reasons for drop in MCH services utilization during specific health emergencies		
	Zika	Ebola	COVID-19
Antenatal services	• Lack of clarity among dengue, chikungunya and zika. • Geographical distance to health facilities • Out of pocket expenditure. • Diagnostic services lacking or not in a timely manner.	• Media disseminating inaccurate/excessive information about the disease • Geographical distance to health facilities • Lack of public transport. • Reluctant to touch them due to insufficient knowledge and training. • Diagnostic services lacking or not in a timely manner. • Providers charging for informal fees	• Fear of infection • Unavailability of staffs • Prioritization to COVID-19 Over pregnancy • Exaggerated information by media • False conception about the origin of the virus. • Perceived threat of being admission if tested positive • Changed protocol created confusion.
Intra-natal services	Not reported	• Geographical distance. • Ambiguity on prevention precaution. • No touch policy. • Staff shortage. • High cost of care • Misconception/Fear regarding infection • Insufficient PPE and basic supplies • Trust in traditional birth attendants.	• Confusion due to Lockdown protocols. • closed government facilities • Inclination to private clinic treatment. • Fear of infection • ‘risk of a Caesarean- Section’ • restriction on accompanying persons • Fear of being isolated if Positive • Lack of affordable maternity facilities
Postnatal services	Not Reported	Not Reported	• Poor access to Health Care Facilities. • Early discharge of postpartum mother • due to fear. • Postponing/ cancelling postnatal services or substitution with virtual services • shortage of healthcare staff in facilities
Family planning services	Not reported	• Lack of Transportation • Lack of privacy • Out of pocket • Lack of resources (water, diagnostic kit) • Restriction on escort • Unclean public toilets. • Shortage of contraceptives/ essential medicine. • Mistrust and misconception resulting in ignorance. • Restricted functional hours. • Geographic distance. • disorganized and difficult admission process (time taking) • long waiting time • Lack of staff & administrative organization • Perceived mistreatment causing dread.	• Telemedicine is an effective way • Cancellation or postponing non- essential services
Child health and Immunization services	Not reported	• Stockout of drugs. • Complications due to delay in obtaining services. • Violence at facilities. • Threat of attack. • Stable coverage due to Vaccination at door step)	• Perceived fear (Penta dose) • Lack of family support • Lockdown, social distance and logistic difficulty(access) • Change in protocol • shortage of manpower, lack of PPE and vaccine supply(Provider). • Infection fear and vaccine hesitancy & social concerns(user) • Altered and delayed vaccination schedules • Expressed “conspiracy theory” and “anti-vaccine sentiment.

## Results

### Search results

**Fig 1:**
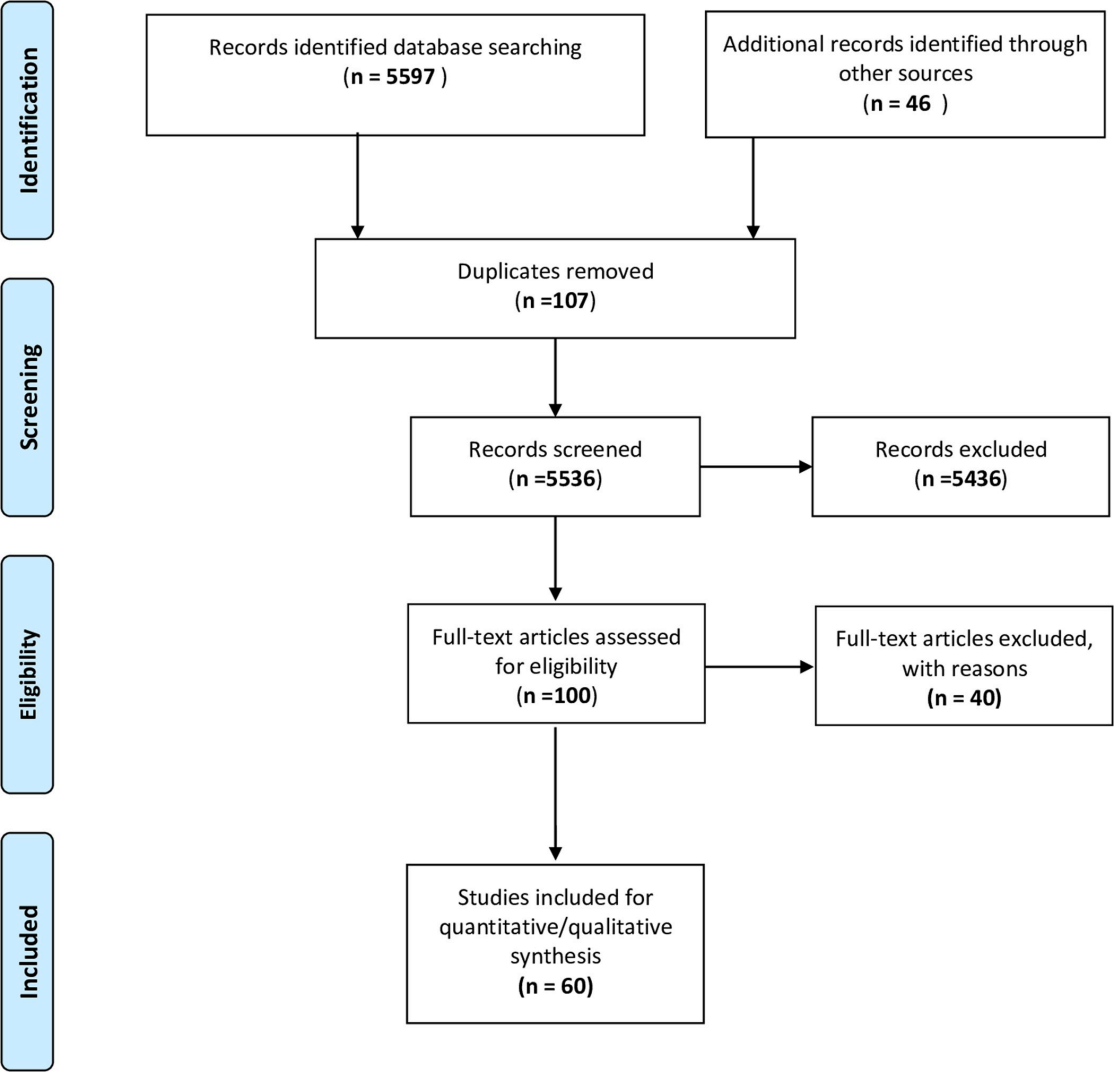
PRISMA flow diagram for the study selection.

Our rigorous and comprehensive search identified 5643 citations including 107 duplicates. Of 5536 citations, 5436 were excluded during the title and abstract screening. Of included potential articles, 40 were rejected during the full-text screening (**S1 File**). A total of 60 studies were finally included in this review. The same is represented using the PRISMA flow diagram (**Fig 1**).

### Study characteristics

**Fig 2:**
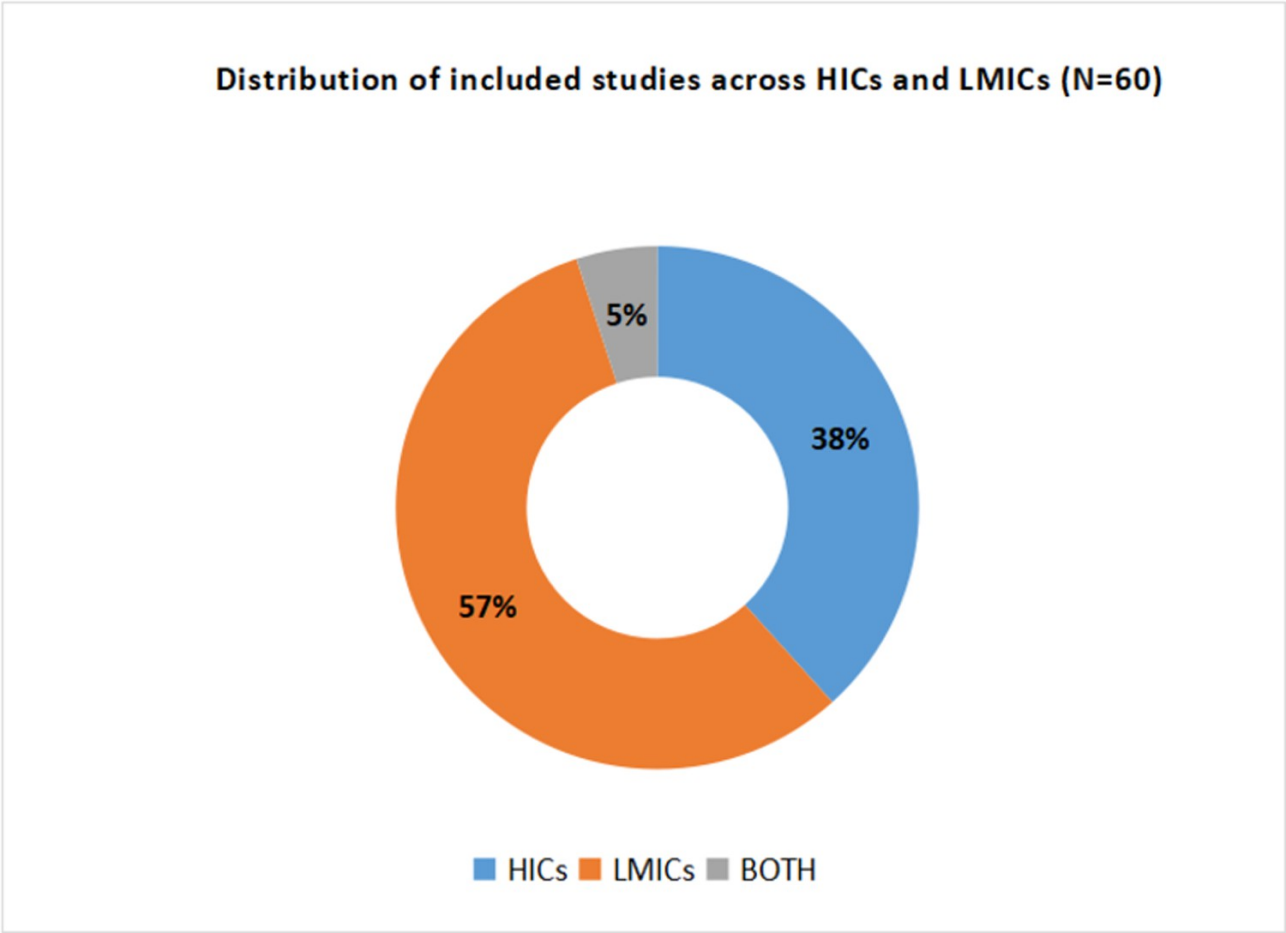
Distribution of included studies across HICs and LMICs.

**Fig 3:**
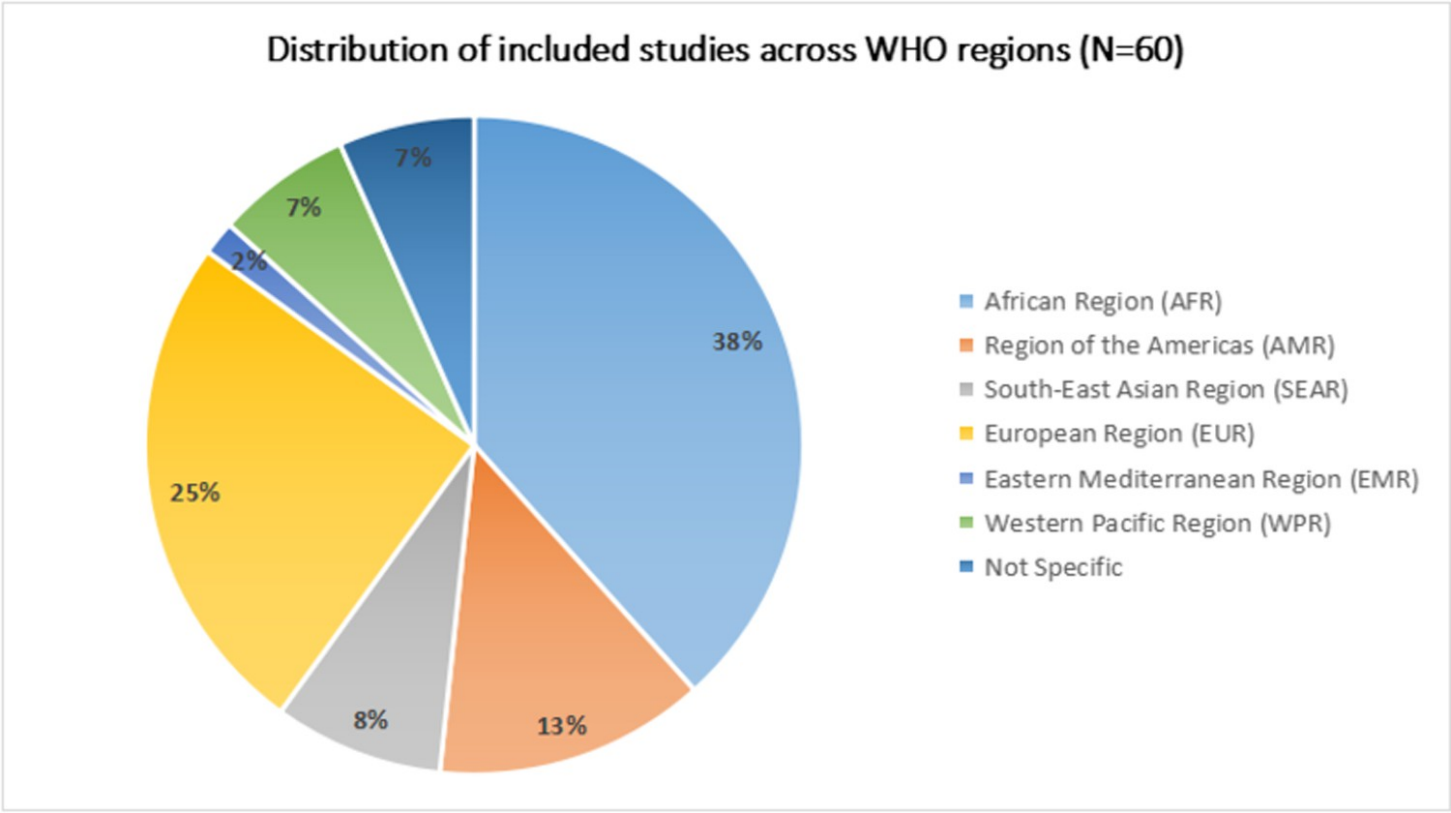
Distribution of included studies across WHO regions.

**Fig 4:**
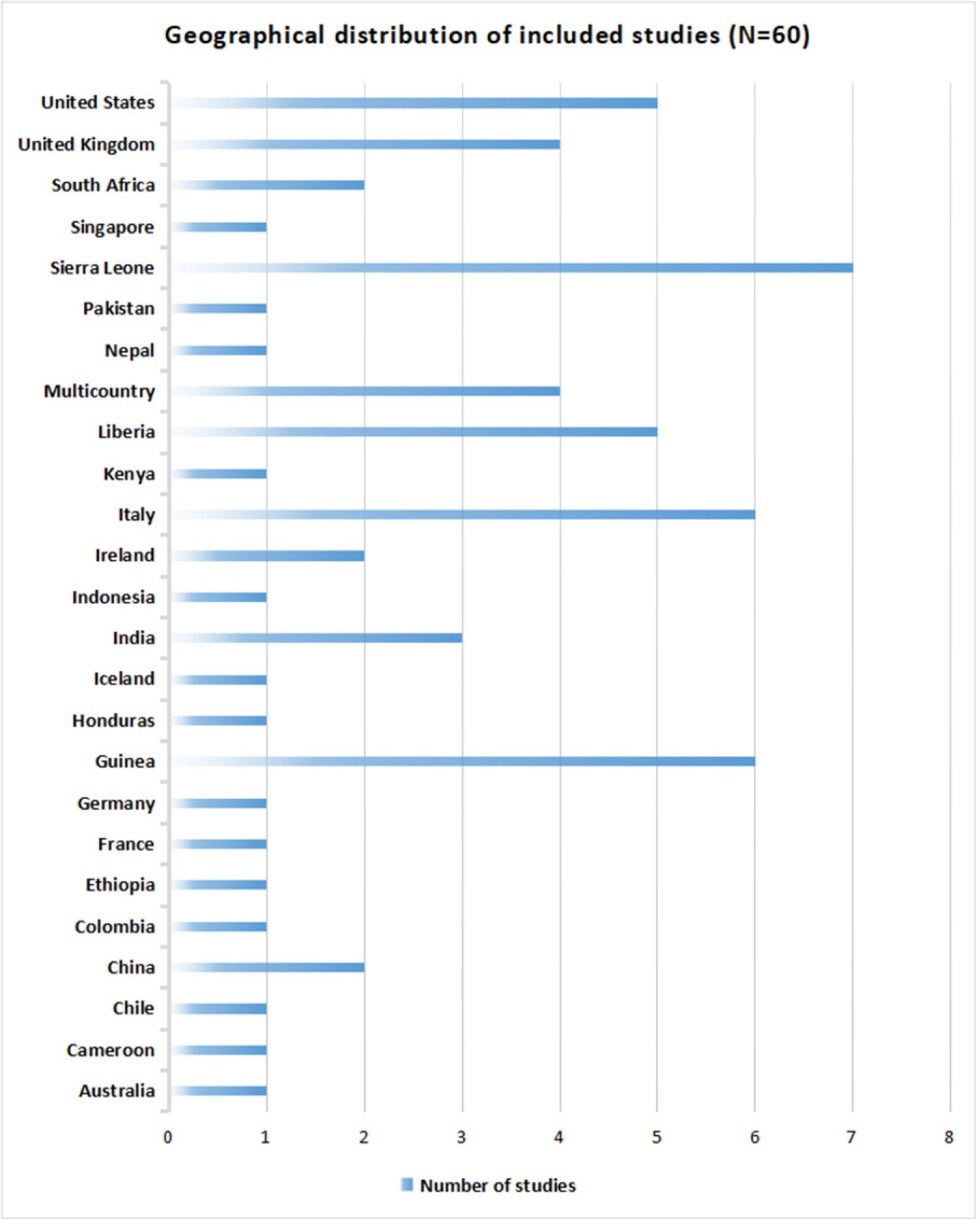
Geographical distribution of included studies.

Detailed characteristics of included studies are provided in the **S2 File**. Among included studies, 11 were qualitative, 8 were mixed methods, and 41 were quantitative. The included studies were from high-income countries (n = 23), LMICs (n = 34) and both (n = 3) (**Fig 2**). Most of the included studies were from the African Region(38%), followed by European Region(25%), Region of the Americas(13%), South-East Asian Region(8%), Western Pacific Region(7%), and Eastern Mediterranean Region(2%) while 7% were not specific to any region (**Fig 3**). Geographically, included studies were widely distributed across the globe comprising Australia, Cameroon, Chile, China, Colombia, Ethiopia, France, Germany, Guinea, Honduras, Iceland, India, Indonesia, Ireland, Italy, Kenya, Liberia, Nepal, Pakistan, Sierra Leone, Singapore, South Africa, United Kingdom, and the United States (**Fig 4**).

The included studies were from healthcare facilities (n = 39), community (n = 5), in both facility and community (n = 3), conducted online mode (n = 6), and based on electronic databases or hospital records (n = 7). The targeted population under study included both MCH service beneficiaries and healthcare providers. However, the number of studies targeting beneficiaries (n = 38) was two times higher than that of health care providers (n = 16), and very few considered both groups (N = 6). Although the search retrieved articles published till January 2021, the included articles were published between 2015 and 2021. Out of 60 studies, 39 emphasized the COVID-19 pandemic, while 18 studies focused on the Ebola epidemic and three on the Zika virus epidemic. Twenty-two studies reported on the interventions or strategies employed to ensure MCH services; however, none reported their effectiveness. Thirty-three of sixty included studies were funded.

### Assessment on quality of study methodology

The quality of each included study was evaluated for its validity, reliability, and results using JBI tools of critical appraisal which used a range of criteria that measured as being “met” or “not met” or “unclear” or “not applicable” and weighted accordingly. Of 60 studies, 37 were weighted above 70% and categorized as high quality, while 19 studies scored between 40–70% (moderate) and four scored below 40%, thus categorized as of low quality. However, articles rated low quality were not excluded due to the scope of study results. The detailed quality assessment of selected studies is presented in the **S3 File**.

Insufficient reporting of outcomes (on WHO RMNCH indicators) and no reporting on the effectiveness of identified interventions in included quantitative studies and heterogeneity made it impractical and inapt to conduct a statistical analysis/meta-analysis. Thus, a narrative approach was used to summarize the outcomes of quantitative studies, further complemented by qualitative findings. The findings are presented according to the WHO RMNCH framework.

#### 1. Reproductive health

*1*.*1*. *Family planning services (FPS)*. The utilization of FPS was affected during the unprecedented events, which were quite evident from the included studies. During the Ebola crisis, the utilization of FPS declined by 51% compared to pre-Ebola [22]. A study mentioned that contraceptives (viz. injectable, oral contraceptive pills, or condoms) and key medications went out of stock during Ebola [22]. Another study documented that utilization of family planning services declined by 6% during the Ebola outbreak [23]. Despite a drop in FPS utilization during the early phases of lockdown, a sharp reversal in FPS service uptake during the post-lockdown phase was reported [24]. Furthermore, distance from the health facility, restricted functioning hours of the facility, long waiting time [25], restrictions to getting inside the hospitals [25], and fear of contracting the infection were significant limiting factors for availing FPS.

#### 2. Maternal and newborn health

*2*.*1*. *Antenatal services (ANC)*. Few studies reported the changes in antenatal service utilization during pandemics. The utilization of prenatal services decreased by about 58% during the Ebola pandemic [26–29]. Conversely, 55.5% of expectant mothers said they missed or delayed their antenatal check-ups during COVID-19 [30]. Three studies revealed that women had attended fewer ANC check-ups than the recommended number during the COVID-19 pandemic [30–32]. Women in the second trimester of pregnancy attended more ANC visits (48%) compared to those in the first and third trimesters of pregnancy (39.5% and 35.2%, respectively) during the COVID-19 pandemic in China [33]. Inappropriateness in the quality of ANC services was reported by 27.8% of Indonesian women as ANC examination was not performed under the scope of ANC service provision by midwives [34]. During Ebola, studies indicated a 22% loss in achieving four or more ANC visits in Liberia, while a COVID-19 related study in Ethiopia revealed a 1.8% loss for the same [23, 27, 30]. In India, hampered transportation (50.9% respondents) and fear of infection (33.4% respondents) were primary reasons for missing ANC check-ups [32]. Similarly, an Ethiopian study found that missed ANC visits were associated with a lack of travel means during lockdown (28%), fear of infection (56.8%), stay-at-home instructions (17%), and redirection of maternal health services/personnel (33%) [30]. Higher demand for virtual patient consultation during the pandemics was noticed. Another study from China reported that 59.6% of beneficiaries used remote consultation, and 42.2% requested fewer in-person ANC visits [33]. However, 50% of women used telemedicine in the US, and 10.7% of face-to-face appointments were reduced during COVID-19 [35]. There were just two studies that looked at how tetanus immunization rates changed among pregnant women during pandemics. During the COVID-19 pandemic, a study in Ethiopia [30] reported a 38.3% drop in TT 1^st^ dosage and a 59.6% drop in TT 2^nd^ dosage; however, during Ebola in Sierra Leone, a 15% dip in TT 2^nd^ dosage was observed. Similarly, a 28.8% dip was observed in overall TT vaccination during the lockdown in Pakistan [36].

Likewise, the qualitative findings of the reviewed articles reported a decline in ANC service uptake. This decline was attributed to numerous factors including inadequate information, geographical barriers, high cost of institutional care, dread of infection, a lack of diagnostic services, and shortage of healthcare workers [25, 37–46]. Beneficiaries in Zika-related studies claimed that they were aware of the potentially harmful effects of the infection on the fetus and preventive measures [25]. On the other hand, during Ebola and COVID-19, beneficiaries were apprehensive about the transmission of disease and pointed out that the media was disseminating misinformation or excessive information about the pandemic [39, 46]. Various misconceptions such as “the deliberate creation of virus by medical workers for financial benefit” or “to create the need for more drugs” [37] further contributed to people’s mistrust of health workers [41]. Most of pregnant women were hesitant to visit health facilities for fear of acquiring the infection, thus missing their ANC visits or accessing facilities only in extreme situations [41–43, 46].

*‘‘When you visited the hospital, you saw that they started bringing Ebola patients and this was the reason you started to fear hospitals." [Gizelis et al. 2017]*

Studies have also reported that other factors for missing ANC visits were the difficulties in reaching out to health facilities due to the distance and the lack of public transport facilities during pandemics [37, 41, 44, 45]. Even those who reached the facility faced difficulties accessing the health services as a priority, and preferences were given for COVID activities. The beneficiaries felt that even the health workers were reluctant to touch them for examination [40, 41, 47]. Furthermore, the facilities were not promptly providing diagnostic services to pregnant women [44].

*‘‘No one wanted to touch a pregnant woman, everyone was afraid. So it was a difficult problem" [Gizelis et al. 2017]*

Healthcare providers reported insufficient knowledge and training on the usage of personal protective equipment (PPE) during the outbreak. As a result, they were terrified to touch the patients. They also perceived that community members have a low level of trust in healthcare providers [39, 40]. Moreover, the frequent changes in hospital protocols during COVID-19 created confusion among the health care providers [48]. So, they tried to avoid face-to-face consultation and preferred virtual consultation [48].

*2*.*2*. *Intrapartum services*. A total of 13 studies focused on how the pandemic influenced intrapartum services. Studies from Guinea, Sierra Leone, and Liberia during the Ebola outbreak revealed a dramatic drop in institutional delivery rates between 9% to 20% [23, 27, 49–51]. However, there was no variation in the percentages of home births. Conversely, a study found a 62% reduction in institutional delivery during peaks of Ebola in one district of Liberia [26]. During Ebola, it was reported that under the most conservative scenario, a decrease in utilization of life-saving health services resulted in 3600 extra stillbirths, maternal and neonatal deaths in 2014–15 [23]. In Guinea, a 14% increase in MMR and a 24% rise in the stillbirth rate are reported during the Ebola epidemic [49]. A study in Monrovia found no significant differences in the proportions between home and facility deliveries; however, there was a considerable shift from the public (31% decline) to private health facilities (increased to 47%) for deliveries [40].

Studies reported that institutional delivery rates fell by nearly half during COVID-19 and other pandemics (Zika and Ebola) [29, 32, 52]. In India, there was a 45% decrease in institutional deliveries and a 7.2% increase in high-risk pregnancies, whereas, in Nepal, a 52.4% decrease in facility births was reported. Furthermore, the neonatal mortality rate rose from 13/1000 live births to 40/1000 live births (p = 0.0022), and similarly, the stillbirth rate rose from 14/1000 live births to 21/1000 live births (p = 0.0002) [32, 52]. A study conducted in South Africa reported a 47% increase in neonatal in-facility mortality due to disruption of health services during COVID-19 [53].

The qualitative articles explained the decrease in institutional deliveries and women’s preference for private facilities. According to studies, women living further away from a healthcare facility were less likely to undergo institutional delivery [40, 45, 49]. Furthermore, many government healthcare facilities were closed during the COVID-19 pandemic, which caused them to seek care in private clinics if they could afford it or give birth at home [41].

*"During COVID-19, there were no delivery services in government, so they chose to seek the services at the private facilities. Private facilities received a high number of mothers; therefore, they hiked the charges which most mothers could not afford." (Luignaah et al. 2016)*

Many women feared that they would be sent to quarantine centers and face exorbitant treatment costs if they tested positive [41, 45, 49]. The majority of women believed that going to the facility would put them at more risk of contracting the infection, which could harm their baby. Moreover, if they are detected to be COVID-19 positive, they may not deliver their child normally or will be separated from their child [41, 43, 44, 46]. Another noteworthy finding was a restriction for birth companions to accompany the women during labor [42–44, 46]. Even for women who visited hospitals, it was studied that due to the “No-touch policy”, the healthcare workers did not use partographs or fetoscopes during labor, jeopardizing the quality of care [49, 54]. Other reported attributing factors to the quality of intrapartum care included shortage of qualified staff and basic supplies such as thermometers and gloves [39] and insufficient or inappropriate size PPE [20, 42].

*2*.*3*. *Postnatal services*. The influence of pandemics on postnatal services was evident in three studies. A drop of 13–22% in postnatal attendance was reported in Sierra Leone during the Ebola epidemic [23, 49]. On the other hand, it was found that postnatal attendance remained stable during COVID-19 in South Africa [24].

The qualitative evidence suggests that the decreased utilization of postnatal services was due to poor access to healthcare services, a shortage of healthcare staff in facilities and a short hospital stay [42]. Postnatal mothers were discharged earlier from health facilities due to fear of infection transmission [44, 46]. During the COVID-19 pandemic, postnatal outpatient services were either cancelled or postponed [42] and, it was substituted with virtual consultation (which was not accessible to all) [43, 42].

*“The lack of time and staff will lead to mothers and babies going home with very little feeding support or knowledge which will have a short and long term impact on their health and ability to deal with infections” [Semaan et al. 2020]*

#### 3. Child health

During the COVID-19 crisis, a significant decrease in acute respiratory infection (ARI) and diarrhea cases were reported among under-5 children. Studies reported a 66–92% decrease in diarrhoea cases, while a 10.3% to 89% decline in ARI was reported [22, 53, 55, 56]. A drop in utilization of emergency department (ED) services was documented. The reasons for reluctance among parents to consult health services were fear of catching infections, especially in health institutions (96%), strict compliance with confinement (30.7%), and financial difficulties (13.9%) [57]. A decline of 46% - 83.8% in pediatric ED admission was demonstrated at the pediatric ED during the COVID-19 pandemic [51, 55, 56, 58–60]. A 36% decline in under-5 children consultation was reported during the Ebola outbreak at the primary healthcare level [61].

*3*.*1*. *Immunization*. A fall in the immunization uptake and reduced compliance with vaccination among parents, was reported due to the COVID-19 crisis [31, 62, 63]. A decline for BCG vaccination reported in the range of 21–56.6% [27, 29, 36], 0.4% to 40% decline in Pentavalent [22, 27, 62, 64], 51% decline for polio doses [27, 65], 5–30% decline in measles first dose [53, 65] and 25.6% to 73.6% drop in measles, mumps and rubella uptake were reported irrespective of the settings (public or private) [64]. Vaccination coverage was stable among a few communities during Ebola due to the doorstep vaccination strategy [66]. Qualitative findings attributed multiple factors to a decline in immunization uptake. Perceived fear of catching infection affected BCG and Pentavalent vaccination; also women failed to turn up to session sites due to lack of familial support [41].

*"Just near our facility here, we came across a mother who had her child miss the immunization for nine-month because her husband did not allow that to happen." [Lusambili et al. 2020]*

Other commonly reported barriers to accessing immunization services included lockdown-related mobility restrictions, inadequate staff, infrastructure, and logistic issues [67]. In LMICs, barriers were mostly skewed towards vaccine inadequacy, vaccine hesitancy, and calling-off clinics. In high-income countries, fear of contracting COVID-19 and changes in management norms like shifting towards virtual consultations attributed to a decline in vaccine uptake [67].

### Interventions/strategies for MCH services

Various studies reported on implementing interventions (**Table 2**) or strategies (**Table 3**) to improve maternal and child health services irrespective of the type of crisis but none of them assessed their effectiveness.

**Table 2:** Interventions to ensure MCH services with quality care during pandemic related health emergencies (Zika, Ebola and COVID-19).

Domain	Intervention	Level		Ensure Quality	Ensure Services	Effectiveness
		*Community Facility*				
General	Telemedicine Telephonic consultation/Telecommunication	✔	✔	✔	✔	These interventions were recommended but the effectiveness was not reported in included studies.
Maternal	Telemedicine	✖	✔	✔	✔	
	Awareness and education activities	✔	✖	✔	✔	
	Incentivizing and rewarding	✔	✖	✔	✔	
	Training and engagement of TBAs	✔	✖	✔	✔	
Child health and immunization	Awareness and education activities	✔	✔	✔	✔	
Family Planning	Telemedicine	✖	✔	✔	✔	
	Awareness and education activities	✔	✖	✔	✔	

**Table 3:** Strategies to ensure MCH services with quality care during pandemic related health emergencies (Zika, Ebola and COVID-19).

Domains	Strategies	Rationale
**General measures**	Postponing or cancelling non-essential consultations	minimize crowding
	Reorganizing the flow of patient	prevent cross- infection among patient
	Strict screening and testing protocol for patients and attendant	identify infected person and prevent cross infection
	Signposts to guide the patients to screening and triaging areas	to prevent overcrowding
	Proper staff management using a weekly roster	maintaining the rhythm of health services
**Antenatal services**	Visits on appointment	to avoid crowding and prioritizing only emergency cases
**Intra-natal services**	A dedicated area for infected women care	to prevent cross-infection
	Emergent Caesarean Section for critically ill COVID positive women	to prevent maternal and fetal mortality.
**Postnatal services**	Speed up post-delivery patient discharge	to reduce period of exposure to infectious environment
**Family planning**	No strategies reported	Not reported
**Child health services**	Kangaroo mother care for sick infants	reduce the risk of death by 65 times from COVID19
**Immunization**	No specific strategy reported	Not reported

***Telemedicine*** utilization has been reported to have increased by 22.4% during the pandemic compared to normal situations [68–70]. An upsurge in virtual prescriptions (55.6%) and consultation (127%) was reported from the primary health centers during the COVID-19 crisis compared to normal conditions [71]. About 79.5% of healthcare providers (HCPs) strongly agreed that telemedicine was a successful technique for contraceptive counseling, with 84% of HCPs agreeing to continue with this technology even after the pandemic [68, 72]. Upsurge in telemedicine uptake by the women for various services viz. surgical abortion (41.7%; 2.9% prior to COVID-19), medical abortion (32.1%; 17.1% prior to COVID-19), prenatal care (19.2%; 5.7% prior to COVID-19), contraception (15.4%; 25.7% prior to COVID-19) has been reported [69]. Patient satisfaction with telemedicine increased by 8.7% for its access, but it decreased for the payment process by 5.3% and infrastructure requirements by 3.4%. Although beneficial, challenges have been reported concerning its mode of functioning, ensuring accessibility to patients, scheduling, and resource requirements [73].

***Telephonic consultation/Telecommunication*** was reported to be another mode for ensuring MCH care during crises [42, 74]. It imparted more comprehensive coverage with less travel, so it was adopted for consultation with lesser hesitancy [68]. Concern over poor connectivity and injustice among those not acquainted with the technologies were reported for causing disparity in service uptake or delivery [68]. The unavailability of technology for virtual (video) consultation led to the uptake of telecommunication as an alternative to serving the purpose[75]. HCPs appreciated and preferred the virtual consultation mode.

*"I am currently doing virtual visits and my mommies [clients] are liking it. They are liking that we take them into consideration and not going and spreading germs from one house to the other. Some mommies have shared and voiced that they would not allow me to come to visit them if I came in person because they are not the only family I see.–Home Visitor." [Marshall et al. 2020]*

***Awareness and educational activitie*s** during post-Ebola gradually enhanced the number of ANC visits, immunization, and family planning care [66]. Engaging pregnant women and health care providers in promoting activities sensitized the community to utilize hospital-based care [66]. To manage the patients’ flow and infection transmission, providers reported a shift in their service delivery methods through rigorously following sanitization measures [22] and allocating dedicated areas for the infected cases [76].

***Incentivizing and rewarding*** were suggested by the healthcare workers and community members towards encouraging ANC registration and utilization of facility care during the Ebola epidemic [66].

***Training and engaging*** the traditional birth attendants [TBAs] for community sensitization and mobilization for institutional care was an important measure, as they were the primary contact point of care and most trusted by the community [66]. During the Ebola outbreak, TTMS (trained traditional midwives) and TBAs were designated as the primary point of care for pregnant and delivering women, carrying out the majority of births and continuing to do so even after the epidemic was over [38].

Along with the interventions mentioned above, various other strategies were adopted to ensure MCH services like postponing or cancelling non-essential consultations through triage, reorganizing the flow of patients (to prevent cross-infection) [63, 69, 70], avoiding physical consultation, and practicing virtual consultation [43]. Visit on appointment was preferred by 87.7% of pregnant women (to avoid crowding), while 59.6% wanted remote consultation and 45.3% wished for joining online maternity schools [33]. It is estimated that a 50% reduction in kangaroo mother care (KMC) coverage can increase a 2.3% - 4.6% neonatal mortality across 127 nations [77]. For low birth weight neonates, KMC was recommended, as it is 65 times more beneficial than the risk of death from COVID-19 [77].

A study [48] reported that 70.9% of HCPs chose to provide ANC services via video conference/phone. However, 79.1% planned to reduce ANC attendance, 44.6% changed the antenatal screening pathway, 39.9% changed the protocol to speed up post-delivery patient discharge, and 78.4% decided on a dedicated area for COVID-19 positive women’s care. During COVID-19, the length of hospital stay (LOS) was reduced by 30% by expediting early discharge for uncomplicated births (LOS after vaginal birth and cesarean section was 14–24 hours and 23–48 hours, respectively) [24, 78]. Qualitative findings suggest suspending of elective gynecological services, reducing post-delivery hospital stay, and restricting ANC meetings as adopted strategies during the COVID pandemic [48].

**Fig 5:**
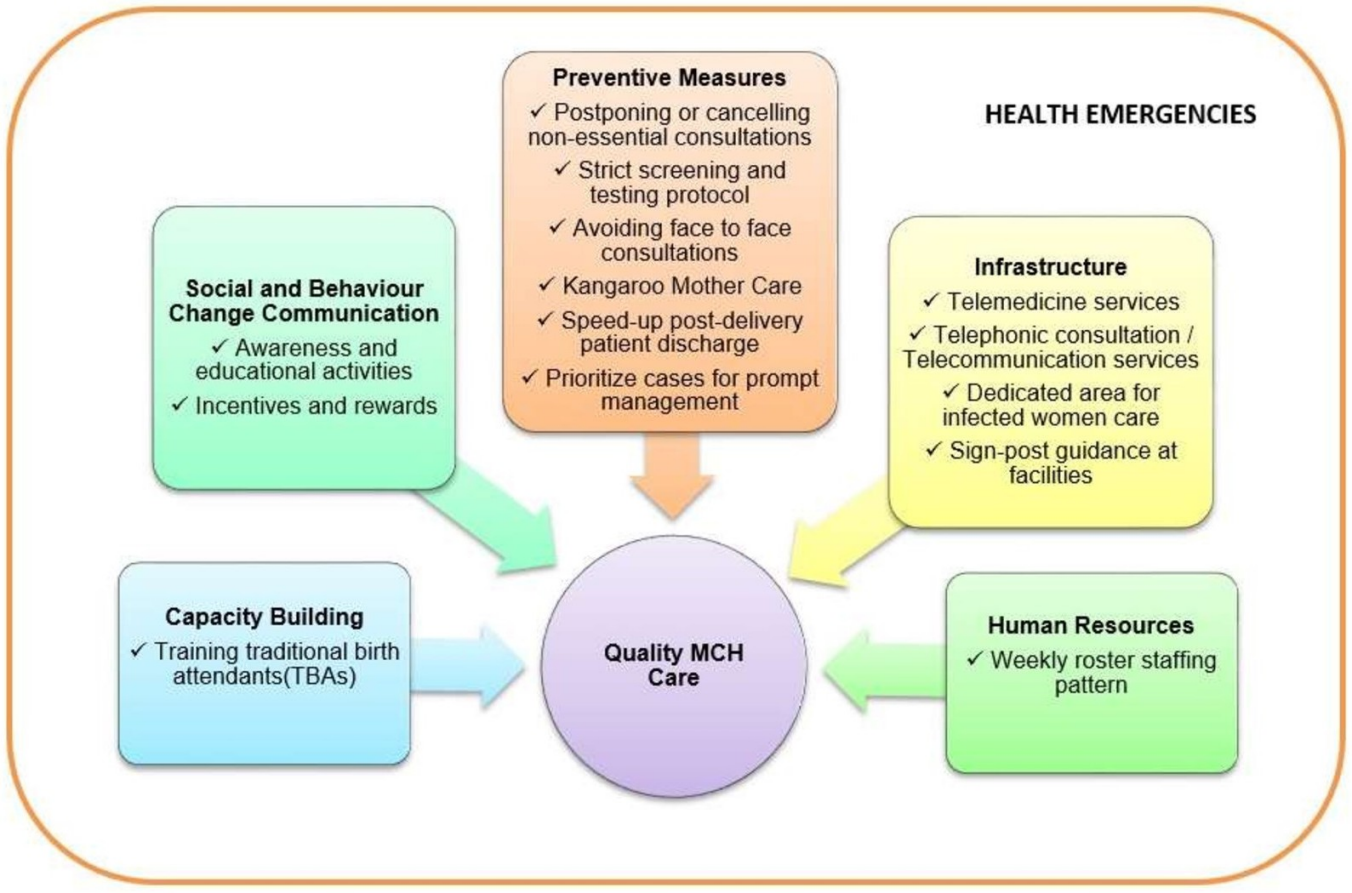
Framework for ensuring quality MCH care services during pandemic related health emergencies (Zika, Ebola and COVID-19).

Other strategies adopted during the COVID-19 pandemic include strict screening and testing for patients, restricting attendants, virtual follow-up of cases [76], patient triage, and screening before the appointment [43, 48]. In some facilities, signposts were used to guide the patients to screening and triage areas [42]. Proper staff management used a weekly roster to maintain the rhythm of services by dedicated team and space to keep a check on existing pandemics [76]. Based on the above interventions and strategies, a framework is developed (**Fig 5**) for ensuring MCH care during emergencies.

## Discussion

This review found that all study settings had positive trends in MCH indicators before pandemics despite several unmet goals. However, these improvements were halted and even reversed during the pandemics. A significant decline was observed across all maternal and child-related health services. It was evident from this review that the utilization of MCH services was hampered due to various attributes in the event of pandemics.

Family planning service utilization was disproportionately affected during health emergencies(Zika, Ebola, and COVID-19). The unmet need for family planning services was further exacerbated due to reduced access to family planning methods due to the pandemic. According to the UNFPA report, COVID-19 containment measures were supposed to prevent approximately 47 million women from using birth control methods in 114 LMICs, resulting in 7 million unintended pregnancies [79]. Hence, to avoid unwanted pregnancies and prevent related maternal mortality and morbidity, each country needs to have a comprehensive health system that ensures family planning services during pandemics. Alternative models of outreach services, such as a home visit by frontline staff, telemedicine, and involvement of private institutions, could be planned, ensuring adequate protection for HCPs [80]. Disease outbreaks often negatively affect women’s healthcare to a broader extent. According to a systematic review, the increased adverse maternal consequences result from of health system incompetence and their inability to cope with the pandemic [81]. Thus, it is strongly advised that adequate staffing for MCH care needs to be prioritized. Training and capacity building of frontline workers should be emphasized to provide safe maternity care during emergencies. Moreover, a separate task force should be formulated to keep frontline workers free to provide maternal health services [82]. Promoting safe and accessible maternity care is essential even during pandemics by having an efficient and sustainable MCH care model to prevent preterm births, stillbirths, and maternal mortality [81].

A significant drop in utilization of pediatric ED services and immunization services during pandemics is also demonstrated. A decline in ARI and diarrhea cases was found among under-5 children during the pandemic. This significant fall could be attributed to improved hand hygiene practices and reduced outdoor exposure due to lockdown and isolation [83]. Factors such as limited information about the availability of health services at facilities, prevailing rumors about the pandemic, and fear of contracting the infection reduced the uptake and utilization of routine MCH care [5]. A study conducted in Pakistan found that around 25,000 polio workers were reallocated to assist with the COVID-19 response [84]. Similarly, measles immunization campaigns in 23 countries were halted, affecting nearly 80 million eligible children during COVID-19 [85]. The cessation of immunization may result in the global spread of vaccine-preventable diseases. Vaccinations are time-sensitive, and if children are not vaccinated within the due time, they will miss out on the benefits of lifelong immunity, exposing the whole cohort to vaccine-preventable diseases [84].

The evidence suggests that telehealth and virtual platforms have the potential to aid in response to large-scale outbreaks and emergencies. Telemedicine was found to have quite a similar impact to face-to-face consultation [86]. Allowing patients and their families to receive telehealth care at home instead of a healthcare facility has improved access to care [87]. Telehealth allows patients and clinicians to communicate faster, enabling self-management and modifications to avoid inpatient care and promoting access to care [87]. However, there may not be sufficient resources for providing telemedicine and virtual patient care at certain medical facilities in some countries. With adequate funding and proper planning, telemedicine could be an instrumental model in the continuity of MCH healthcare services in future emergencies.

Appointment-based visits for routine care reduced the patient loads at the health facilities. Beneficiaries, as well as providers, have shown their acceptance of this strategy. Triage has also been reported to positively influence the utilization and delivery of the services through postponing or canceling trivial patients [88]. Modifications to existing protocols/guidance for service delivery effectively increase healthcare uptake and service delivery.

### Study limitation

Although we tried to explore all available databases, some (CINAHL, Web of Science, others) went unexplored due to limitations to access. Most of the included studies were in the LMICs context; thus, results may be interpreted accordingly. Despite the rigorous review, there was limited evidence on the effectiveness of interventions in the pandemic(Zika, Ebola, and COVID-19) context, which also restricted us from conducting statistical analysis/ meta-analysis.

### Policy implication

The study findings have far-reaching implications. Virtual clinics/web-based consultations have emerged as a novel model for treating patients remotely in emergencies like pandemics. As a result, more rigorous studies are required to assess its long-term impact on patients, healthcare workers, and the healthcare system. Moreover, there is a need to rebuild the trust among beneficiaries in health services by addressing their fear of contracting an infection through adequate protection. Building the trust and confidence of mothers (beneficiaries) and the health care workers, addressing transportation-related challenges to health facilities during pandemics are critically important. Governments might also invest in employing more health professionals and incentivize current workers to boost their work efficiency morally for ensuring quality maternal and child health services. Moreover, Community health workers played a pivotal role in providing MCH services even in the event of pandemics. Hence, their training and capacity building should be prioritized.

## Conclusion

The provision of MCH services has been an uphill battle globally due to the unique situations created by pandemics. However, it allows us to re-evaluate the lacunae in the service provision of the existing health system. This systematic review highlights that pandemics have negatively influenced maternal and child health services. Nonetheless, virtual consultation, patient triage, and the development of dedicated COVID maternity centers and maternity schools have emerged as new concepts in ensuring continuity of care during emergencies like pandemics. However, there is a need for evidence about their effectiveness. It is critically important to have appropriate evidence-based interventions for better MCH care utilization.

## Supporting information

S1 Checklist PRISMA checklist. (PDF)

S1 File List of excluded studies with reasons. (DOCX)

S2 File Characteristics of included studies. (DOCX)

S3 File Quality appraisal of included studies. (DOCX)

S1 Data Search strategy. (DOCX)
