# Supplementary material for: Effective interventions to ensure MCH (Maternal and Child Health) services during pandemic related health emergencies (Zika, Ebola, and COVID-19): A systematic review
Source: PLoS One. 2022 May 10;17(5):e0268106. doi: 10.1371/journal.pone.0268106 (PMC9089853; doi:10.1371/journal.pone.0268106)
Supplement: S1 File — (DOCX) [file pone.0268106.s002.docx]

Excluded studies with reasons

| **S.N.** | **Author** | **Reason for exclusion** |
| --- | --- | --- |
| 1 | Abbas et al. 2020[1] | risk benefit analysis |
| 2 | Albuquerque et al. 2019[2] | explore perspective regarding health services for children diagnosed with Zika syndrome |
| 3 | Alex et al. 2020[3] | viewpoint |
| 4 | Alsharaydeh et al. 2020[4] | methodological issue |
| 5 | Buler et al. 2020[5] | preprint article |
| 6 | Derya et al. 2021[6] | not in context of service utilization |
| 7 | Forero‐Martínez et al. 2020[7] | reports factors affecting implementation of action to address Zika effect on reproductive and sexual rights of women |
| 8 | Gibelin et al. 2020[8] | Preprint article |
| 9 | Gomez-Roas et al. 2021[9] | Poster supplement |
| 10 | Gonzalez-Timoneda et al. 2020[10] | preprint article |
| 11 | Jiang et al. 2021[11] | not directly relevant to scope of study |
| 12 | Kimani et al. 2020[12] | review |
| 13 | Lebel et al. 2020[13] | mental health issue |
| 14 | Luquero et al. 2009[14] | not in context of service utilization/delivery |
| 15 | Mahajan et al. 2020[15] | unclear study design |
| 16 | Menezes et al. 2020[16] | not in context of service utilization/delivery |
| 17 | Narla et al. 2020[17] | Viewpoint |
| 18 | Ngo et al. 2021[18] | full text not available |
| 19 | Nosratabadi et al. 2020[19] | Do not explain much about service utilization |
| 20 | Obeidat et al. 2020[20] | explain about awareness regarding risk and preventive measures for covid-19 |
| 21 | Ogundele et al. 2020[21] | preprint article |
| 22 | Pires et al. 2020[22] | Preprint article |
| 23 | Raoul et al. 2010[23] | modeling study |
| 24 | Ravaldi et al. 2020[24] | psychological impact of COVID-19 on maternal health |
| 25 | Raven et al. 2018[25] | Do not explore impact on maternal and child health services |
| 26 | Roberton et al. 2020[26] | primarily focus on paediatric surgery |
| 27 | Rochon et al. 2021[27] | Poster presentation |
| 28 | Sagaram et al. 2021[28] | Poster supplement |
| 29 | Saiman et al. 2020[29] | article in press |
| 30 | Shikuku et al. 2020[30] | preprint article |
| 31 | Stamm et al. 2020[31] | Pregnant Rheumatology outpatients |
| 32 | Steven et al. 2020[32] | do not directly relate with objective |
| 33 | Takemoto et al. 2020[33] | Full text not available |
| 34 | Talati et al. 2021[34] | Poster supplement |
| 35 | Temesgen et al. 2020[35] | preprint |
| 36 | Temesgen et al. 2020[36] | preprint article |
| 37 | Turrentine et al. 2020[37] | commentary |
| 38 | Wenham et al. 2021[38] | abortion services and family planning excluded |
| 39 | Williams et al. 2020[39] | methodological issue |
| 40 | Wong et al. 2016[40] | Poster supplement |

**References for excluded studies:**

1. Abbas K, Procter SR, van Zandvoort K, Clark A, Funk S, Mengistu T, et al. Routine childhood immunisation during the COVID-19 pandemic in Africa: a benefit–risk analysis of health benefits versus excess risk of SARS-CoV-2 infection. Lancet Glob Heal. 2020;8:e1264–72.

2. Albuquerque MSV, Lyra TM, Melo APL, Valongueiro SA, Araújo TVB, Pimentel C, et al. Access to healthcare for children with Congenital Zika Syndrome in Brazil: Perspectives of mothers and health professionals. Health Policy Plan. 2019. p. 499–507.

3. Peahl AF, Smith RD, Moniz MH. Prenatal care redesign: creating flexible maternity care models through virtual care. Am J Obstet Gynecol. Elsevier; 2020;223:389.e1-389.e10.

4. Alsharaydeh I, Rawashdeh H, Saadeh N, Obeidat B, Obeidat N. Challenges and solutions for maternity and gynecology services during the COVID-19 crisis in Jordan. Int J Gynecol Obstet. I. Alsharaydeh, Department of Obstetrics and Gynecology, Faculty of Medicine, King Abdullah University Hospital, Jordan University of Science and Technology, Irbid, Jordan; 2020;150:159–62.

5. Buler M, Pustułka P. Pregnancy and Childbirth During the Covid-19 Epidemic in Poland : Qualitative Evidence From Expert Interviews . BMC Pregnancy Childbirth. 2020;1–20.

6. AKSOY DERYA Y, ALTIPARMAK S, AKÇA E, GÖKBULUT N, YILMAZ AN. Pregnancy and birth planning during COVID-19: The effects of tele-education offered to pregnant women on prenatal distress and pregnancy-related anxiety . Midwifery. 2021;92:102877.

7. Forero-Martínez LJ, Murad R, Calderón-Jaramillo M, Rivillas-García JC. Zika and women’s sexual and reproductive health: Critical first steps to understand the role of gender in the Colombian epidemic. Int J Gynecol Obstet. M. Calderón-Jaramillo, Asociación Profamilia, Bogotá, D.C, Colombia; 2020;148:15–9.

8. Gibelin K, Agostini A, Marcot M, Piclet H, Bretelle F, Miquel L. COVID-19 impact in abortions’ practice, a regional French evaluation. J Gynecol Obstet Hum Reprod. L. Miquel, Department of Obstetrics, Gynecology and Reproductive Medicine, Pôle Femmes Parents Enfants, AP-HM La Conception University Hospital, 147 bd Baille, Marseille, France; 2021;50.

9. Gomez-Roas M, Davis KD, Leziak K, Jackson J, Williams BR, Feinglass JM, et al. 227 Postpartum during a pandemic: challenges of low-income women with healthcare interactions during COVID-19. Am J Obstet Gynecol. 2021;224:S150.

10. González-Timoneda A, Hernández Hernández V, Pardo Moya S, Alfaro Blazquez R. Experiences and attitudes of midwives during the birth of a pregnant woman with COVID-19 infection: A qualitative study. Women and Birth. 2020;

11. Jiang H, Jin L, Qian X, Xiong X, La X, Chen W, et al. Maternal mental health status and approaches for accessing antenatal care information during the COVID-19 Epidemic in China: Cross-sectional study. J Med Internet Res. H. Jiang, School of Public Health, Fudan University, 138 Yixueyuan Road, Shanghai, China; 2021;23.

12. Kimani RW, Maina R, Shumba C, Shaibu S. Maternal and newborn care during the COVID-19 pandemic in Kenya: Re-contextualising the community midwifery model. Hum Resour Health. Human Resources for Health; 2020;18:3–7.

13. Lebel C, MacKinnon A, Bagshawe M, Tomfohr-Madsen L, Giesbrecht G. Elevated depression and anxiety symptoms among pregnant individuals during the COVID-19 pandemic. J Affect Disord. 2020;277:5–13.

14. Luquero FJ, Hernán García C, Eiros Bouza JM, Castrodeza Sanz J, Sánchez-Padilla E, Simón Soria F, et al. Profile of paediatric admissions and emergencies during an epidemic period of rotavirus in Valladolid [Spain]. Utility of a predictive model. Gac. Sanit. 2009. p. 58–61.

15. Mahajan NN, Pednekar R, Patil SR, Subramanyam AA, Rathi S, Malik S, et al. Preparedness, administrative challenges for establishing obstetric services, and experience of delivering over 400 women at a tertiary care COVID-19 hospital in India. Int J Gynecol Obstet. 2020;151:188–96.

16. Menezes MO, Takemoto MLS, Nakamura-Pereira M, Katz L, Amorim MMR, Salgado HO, et al. Risk factors for adverse outcomes among pregnant and postpartum women with acute respiratory distress syndrome due to COVID-19 in Brazil. Int J Gynecol Obstet. M. Nakamura-Pereira, Instituto Nacional de Saúde da Mulher, da Criança e do Adolescente Fernandes Figueira, Fundação Oswaldo Cruz, Rio de Janeiro, RJ, Brazil; 2020;151:415–23.

17. Narla NP, Surmeli A, Kivlehan SM. Agile application of digital health interventions during the covid-19 refugee response. Ann Glob Heal. A. Surmeli, Medical Rescue Association of Turkey (MEDAK), Istanbul, Turkey; 2020;86:1–5.

18. Ngo TM, Rogers B, Patnaik R, Jambai A, Sharkey AB. The Effect of Ebola Virus Disease on Maternal and Child Health Services and Child Mortality in Sierra Leone, 2014–2015: Implications for COVID-19. Am J Trop Med Hyg. 2021;2014–5.

19. Nosratabadi M, Sarabi N, Masoudiyekta L. A case report of vaginal delivery at home due to fear of covid-19. Iran J Psychiatry. M. Nosratabadi, Department in Midwifery, School of Nursing and Midwifery, Dezful University of Medical Sciences, Dezful, Iran; 2020;15:366–9.

20. Obeidat N, Saadeh R, Obeidat M, Khasawneh W, Khader Y, Alfaqih M. Perceptions of obstetricians and pediatricians about the risk of COVID-19 for pregnant women and newborns. Int J Gynecol Obstet. W. Khasawneh, Department of Pediatrics and Neonatology, Jordan University of Science and Technology, Irbid, Jordan; 2020;150:306–11.

21. Ogundele IO, Alakaloko FM, Nwokoro CC, Ameh EA. Early impact of COVID-19 pandemic on paediatric surgical practice in Nigeria: A national survey of paediatric surgeons. BMJ Paediatr Open. I.O. Ogundele, Olabisi Onabanjo University Teaching Hospital, Sagamu, Ogun, Nigeria; 2020;4.

22. Pires P das N, C M, A A, J M, M M, R S, et al. Covid-19 pandemic impact on maternal and child dealth services access in Nampula, Mozambique: A mixed methods research. Res Sq. 2020;1–10.

23. Nap RE, Andriessen MPHM, Meessen NEL, Albers MJIJ, Van Der Werf TS. Pandemic influenza and pediatric intensive care. Pediatr Crit Care Med. R. E. Nap, Directorate of Medical Affairs, Quality and Safety, Beatrix Children’s Hospital, Groningen, Netherlands; 2010;11:185–98.

24. Ravaldi C, Wilson A, Ricca V, Homer C, Vannacci A. Pregnant women voice their concerns and birth expectations during the COVID-19 pandemic in Italy. Women and Birth. 2020;

25. Raven J, Baral S, Wurie H, Witter S, Samai M, Paudel P, et al. What adaptation to research is needed following crises: A comparative, qualitative study of the health workforce in Sierra Leone and Nepal. Heal Res Policy Syst. J. Raven, Department of International Health, Liverpool School of Tropical Medicine, Liverpool, United Kingdom; 2018;16.

26. Roberton T, Carter ED, Chou VB, Stegmuller AR, Jackson BD, Tam Y, et al. Early estimates of the indirect effects of the COVID-19 pandemic on maternal and child mortality in low-income and middle-income countries: a modelling study. Lancet Glob Heal. 2020;8:e901–8.

27. Rochon M, Christman K, Rochon A, Prutsman W, Quiñones JN. 287 Impact of the COVID-19 pandemic on maternal fetal medicine outpatient health care delivery platforms. Am J Obstet Gynecol. 2021;224:S187–8.

28. Sagaram D, Eisenberg R, Karkowsky CE. 963 Can telemedicine improve postpartum access to care? Am J Obstet Gynecol. Elsevier; 2021;224:S598.

29. Saiman L, Acker KP, Dumitru D, Messina M, Johnson C, Zachariah P, et al. Infection prevention and control for labor and delivery, well baby nurseries, and neonatal intensive care units. Semin Perinatol. 2020;44:151320.

30. Shikuku DN, Nyaoke I, Gichuru S, Maina O, Eyinda M, Godia P, et al. Early indirect impact of COVID-19 pandemic on utilization and outcomes of reproductive, maternal, newborn, child and adolescent health services in Kenya. medRxiv. 2020;2020.09.09.20191247.

31. Stamm B, Vitone G, Frey M, Vega J, Salmon J, Crow M, et al. Experience of Pregnant Rheumatology Outpatients from a Tertiary Hospital in New York City during the COVID-19 Pandemic. Arthritis Rheumatol. B. Stamm, Hospital for Special Surgery, Barbara Volcker Center for Women and Rheumatic Diseases, New York, NY, United States; 2020;72:3591–4.

32. Abrams SA, Avalos A, Gray M, Hawthorne KM. High Level of Food Insecurity among Families with Children Seeking Routine Care at Federally Qualified Health Centers during the Coronavirus Disease 2019 Pandemic. J. Pediatr. X. 2020.

33. Takemoto MLS, Menezes MDO, Andreucci CB, Nakamura-Pereira M, Amorim MMR, Katz L, et al. The tragedy of COVID-19 in Brazil: 124 maternal deaths and counting. Int J Gynecol Obstet. M.D.O. Menezes, Medical School of Botucatu, Universidade Estadual Paulista Júlio de Mesquita Filho (UNESP), Botucatu, SP, Brazil; 2020;151:154–6.

34. Talati AN, Mallampati D, Johnson JD, West-Honart A, Vladutiu C, Menard MK. 1009 Provider satisfaction with telehealth for maternity care: a rapid assessment of a national survey. Am J Obstet Gynecol. Elsevier; 2021;224:S625.

35. Temesgen K, Wakgari N, Debelo BT, Tafa B, Alemu G, Wondimu F, et al. Maternal health care services utilization amidst COVID-19 pandemic in West Shoa zone, central Ethiopia. PLoS One. 2021;16.

36. Assefa KT, Gashu AW, Mulualem TD. The impact of COVID-19 infection on maternal and reproductive health care services in governmental health institutions of Dessie town, North-East Ethiopia, 2020 G.C. medRxiv. 2020;2020.09.20.20198259.

37. Turrentine M, Ramirez M, Monga M, Gandhi M, Swaim L, Tyer-Viola L, et al. Rapid Deployment of a Drive-Through Prenatal Care Model in Response to the Coronavirus Disease 2019 (COVID-19) Pandemic. Obstet Gynecol. M. Turrentine, Baylor College of Medicine, 6651 Main Street, Houston, TX, United States; 2020;136:29–32.

38. Wenham C, Abagaro C, Arévalo A, Coast E, Corrêa S, Cuéllar K, et al. Analysing the intersection between health emergencies and abortion during Zika in Brazil, El Salvador and Colombia. Soc. Sci. Med. 2021.

39. Williams K, Ruiz F, Hernandez F, Hancock M. Home visiting: A lifeline for families during the COVID-19 pandemic. Arch Psychiatr Nurs. 2021;35:129–33.

40. Wong RYS, Taylor HG, Sampson JB. Influence of the 2014 West African Ebola epidemic on essential health service utilization in a Liberian district. Ann Glob Heal. R.Y.S. Wong, Johns Hopkins Bloomberg School of Public Health, Baltimore, MD, United States; 2016;82:571–2.
