# Supplementary material for: Effective interventions to ensure MCH (Maternal and Child Health) services during pandemic related health emergencies (Zika, Ebola, and COVID-19): A systematic review
Source: PLoS One. 2022 May 10;17(5):e0268106. doi: 10.1371/journal.pone.0268106 (PMC9089853; doi:10.1371/journal.pone.0268106)
Supplement: S2 File — (DOCX) [file pone.0268106.s003.docx]

| **Sl. No.**  **Characteristics of included studies** | **Author-Year** | **Country** | **Study Setting Facility/Community/Rural/Urban etc.** | **Study Type e.g. Quantitative study/Qualitative study/ Mixed method study** | **Pandemic Type e.g. Zika/ Ebola/ COVID-19 etc.** | **Method of data collection** | **Participants** | **Sample Size** | **Aim/ Objectives** | **Interventions/ Strategies (Yes/ No)** |
| --- | --- | --- | --- | --- | --- | --- | --- | --- | --- | --- |
| 1 | Ahlers-Schmidt et al. 2020 | United States | NA | Quantitative study | COVID-19 | self-administered questionnaires through E-mail | pregnant and postpartum women (infants <12months) who were >15 years | n=114 | to understand knowledge, attitudes and practices of pregnant women and mothers of infants around coronavirus disease 2019 (COVID-19) | No |
| 2 | Ashish et al. 2020 | Nepal | NA | Quantitative study | COVID-19 | participant-level data for pregnant women enrolled in the SUSTAIN and REFINE studies and health worker performance via direct observation | pregnant women | 21763 women | to assess the indirect impact of the COVID-19 lockdown on use of public health facilities for childbirth, quality of intrapartum care, institutional stillbirth rate, and neonatal mortality rate before and during lockdown. | No |
| 3 | Barden-O'Fallon et al. 2015 | Guinea | Facility | Mixed method study | Ebola | Retrospective record review with structured questionnaire | Facility record, Facility Director, RMNCH Service Provider | 5 | to better understand how the delivery and utilization of routine RMNCH services may have been affected by the extraordinary strain placed on the health system and its client population by the Ebola outbreak in Guinea. | Yes |
| 4 | Baumann et al. 2020 | France | all districts in mainland France and overseas territories | Quantitative study | COVID-19 | online self-administered questionnaire | independent midwives | n=1517 | To identify how independent midwives who work in primary care modify their practices at the beginning of pandemic in France | Yes |
| 5 | Bechini et al. 2020 | Italy | Facility | Quantitative study | COVID-19 | Semi structured Online questionnaire | pediatricians | 223 | to evaluate the impact of the COVID-19 epidemic on paediatric vaccinations administered by Tuscan pediatricians, as a proxy of adherence to vaccinations during this epidemic | Yes |
| 6 | Belizan et al. 2020 | Honduras | Facilities | Qualitative study | Zika | semi-structured interviews and focus groups(n=5) | a) women ≥18 years, users of contraception services; b) men ≥18 years, users of contraception services,) b) women ≥18 years receiving PAC; c) men ≥18 years, accompanying partners | N= 136 | to explore and describe users’ knowledge, perceptions and experiences regarding: Zika prevention during pregnancy, contraception and post abortion services | No |
| 7 | Bell et al. 2016 | Liberia | community and facility | Qualitative study | Ebola | semi-structured focus groups | 11 nurses, ten traditional birth attendants (TBAs), four midwives, 28 general community health volunteers, three physician assistants, one community member and one pharmacy dispenser. | 58 participants | To explore healthcare providers' perceptions and reactions to the Ebola Virus Disease (EVD) epidemic. | No |
| 8 | Bianca et al. 2020 | United States | Facility | Mixed method study | COVID-19 | web-based survey | US family planning providers | 172 | The objective of this study is to describe the experience of US family planning providers with the rapid adoption of telemedicine for contraceptive counseling during this period. To identify provider preferences with regards to telemedicine visits, and to outline their recommendations for the future role of telemedicine in contraceptive counseling. | Yes |
| 9 | Bienvenu et al. 2017 | Guinea | NA | Quantitative study | Ebola | from district health committee reports and registers | women of reproductive age (15–45 year old) | 187 094 women | In Guinea, to compare trends in family planning, antenatal care, and institutional deliveries over the period before, during and after the outbreak. | No |
| 10 | Bornstein et al. 2020 | United States | Facility | Quantitative study | Zika | face-to-face in-depth interviews | uncomplicated postpartum women | n=11770 | To report experience with early postpartum discharge to decrease hospital length of stay among low-risk puerperium patients in a large obstetrical service during the COVID-19 pandemic in New York. | Yes |
| 11 | Cella et al. 2020 | Italy | NA | Quantitative study | COVID-19 | survey | NA | 6 pediatric emergency department | To evaluate the effect of the COVID-19 epidemic on paediatric emergency department (ED) attendance in a region of Northern Italy. | No |
| 12 | Chandir et al. 2020 | Pakistan | NA | Quantitative study | COVID-19 | Individual immunization records from real-time provincial Electronic Immunization Registry | NA | 2,787,450 | to measure the reduction in daily immunization rates in Sindh province, report antigen-wise coverage, and drop-out rates for 0–23 month children, identify baseline characteristics associated with drop-outs, and observe the spatial distribution of immunization activity. | No |
| 13 | Chen et al. 2020 | China | community | Quantitative study | COVID-19 | online self-administered questionnaire | All the pregnant women who used the YYT platform | n=2599 | to describe the needs of pregnant women and the contents of online obstetric consultation in representative areas with various severity of the epidemic in China. | Yes |
| 14 | Delamou et al. 2017 | Guinea | NA | Quantitative study | Ebola | Secondary data from monthly facility-based maternal and child health services | women and children | NA | to assess maternal and child health, and more specifically use of antenatal care, institutional delivery, and immunization services, before, during, and after the Ebola virus disease outbreak in the highly affected Forest region of Guinea. | No |
| 15 | Delamou et al. 2017 | Guinea | Facility | Quantitative study | Ebola | standardized self - administered questionnaire | Heath care worker | 299 | to document maternal and child health care workers‘ knowledge, attitudes and practices on service delivery before, during and after the 2014 EVD outbreak in rural Guinea | No |
| 16 | Dopfer et al. 2020 | Germany | tertiary care center | Quantitative study | COVID-19 | NA(routine electronic clinical records) | Paediatric | n = 5,424 | to estimate impact of the pandemic on pediatric emergency healthcare utilization | No |
| 17 | Dynes et al. 2015 | Sierra Leone | Facility ,Community | Qualitative study | Ebola | Focus group discussion | health care worker and pregnant women | 34 Health worker and 27 pregnant and lactating mothers | To understand factors that might have contributed to these declines and to explore approaches to increase use of maternal and newborn health services during the Ebola epidemic.  To assess attitudes and perceptions regarding the risk for Ebola and health facility use among health workers and pregnant and lactating women. | Yes |
| 18 | Enyamaa et al. 2020 | Cameroon | NA | Quantitative study | COVID-19 | online survey | pediatricians | 118 pediatricians | to describe the impact of the COVID-19 pandemic on the clinical activity of pediatricians. | No |
| 19 | Ferrazzi et al. 2020 | Italy | Facility Based | Quantitative study | COVID-19 | Data were collected from the clinical records using a standardized questionnaire. | Pregnant women with COVID-19-confirmed infection who delivered. | 42 | To report mode of delivery and immediate neonatal outcome in women infected with COVID-19. | No |
| 20 | Fumagalli et al. 2021 | Italy | Facility Based | Qualitative study | COVID-19 | Audio-recorded semi structured interviews | mothers (n = 34) who tested positive to COVID-19 and gave birth | 22 | To explore childbearing experiences of COVID-19 positive mothers who gave birth in the months of March and April 2020 in a Northern Italy maternity hospital. | No |
| 21 | Garcia-Huidobro et al. 2020 | Chile | Facility Based | Mixed method study | COVID-19 | open-ended questions |  | Patients:  2962- Receiving Telemedicine 1187: Concurrent Control group  1848: retrospective control group  Providers: 263 physicians | The aim of this study is to report the system-wide accelerated implementation of telemedicine, compare patient satisfaction between telemedicine and in-person visits, and report provider perceptions. | Yes |
| 22 | Garg et al. 2020 | India | Facility | Quantitative study | COVID-19 | Online digital questionnaire | faculties involved in the management and supervision of PHCs | 60 | To determine the primary health care facility preparedness toward the provision of safe outpatient services during the COVID-19 pandemic in India. | No |
| 23 | Gizelis et al. 2017 | Liberia | Urban | Mixed method study | Ebola | data collected from 2 surveys i.e. LDHS 2013 and 2014. Also, FGD and IDI among the women, men and medical personnel and administrator | health care provider, both men and women | qualitative: 23 IDI and 8 FGD  Quantitative: two survey (1073 females in 1st survey, 1573 females in 2nd survey) | Explored how Ebola affected the use of maternal health care services in Monrovia | No |
| 24 | Goyal et al. 2020 | India | Facility Based | Quantitative study | COVID-19 |  | pregnant women admitted during the study period. | 633 | To assess the effects of the COVID-19 pandemic on obstetric care and outcomes. | No |
| 25 | Hector et al. 2020 | Colombia | Municipality | Qualitative study | Zika | face-to-face in-depth interviews | pregnant women infected with zika virus | n=6 | To explore the perceptions and experiences of pregnant women in accessing healthcare services during the epidemic in Colombia during 2015–2016. | No |
| 26 | Hermans et al. 2017 | Sierra Leone | Facility | Quantitative study | Ebola | Health Centre data sheet | The study population included all children (under-five years) presenting for out-patient consultations and b) at the referral hospital level, all children under 15 years presenting at the emergency room and admitted to the hospital wards. | 5223 | we assessed the burden of the 2014 Ebola outbreak on under-five consultations at a primary health center and the quality of care for under-15 children at a Medicines Sans Frontiers (MSF) referral hospital. | No |
| 27 | Homer et al. 2020 | Australia | Facility Based | Mixed method study | COVID-19 | Online survey | Midwives | 103 | To explore the experience of PPMs in relation to the response to planning for the COVID-19 pandemic. | Yes |
| 28 | Indrayani et al. 2020 | Indonesia | Facility Based | Quantitative study | COVID-19 | data collection was done using questionnaire (google form) | All pregnant mothers | 115 | To analyze the ANC services during COVID-19 pandemics | No |
| 29 | Jardine et al. 2020 | United Kingdom | Obstetric units | Quantitative study | COVID-19 | Self-administered questionnaire | Health care worker | 81 | To explore the modifications to maternity services across the UK, in response to the coronavirus disease 2019 (COVID-19) pandemic, in the context of the pandemic guidance issued by the Royal College of Obstetricians and Gynecologists (RCOG), Royal College of Midwives (RCM) and NHS England | Yes |
| 30 | Jensen et al. 2021 | South Africa | District Health Information System(DHIS) data set of the KwaZulu-Natal (KZN) provincial health service. | Quantitative study | COVID-19 | secondary(DHIS) | children under 5 years | NA | To assess the impact of the local COVID-19 outbreak on routine child health services | No |
| 31 | Jones et al. 2016 | Sierra Leone | Facility Based | Quantitative study | Ebola | electronic data collection tool | Antenatal and postnatal women | 1716 | To determine the impact of the Ebola virus epidemic on the availability, uptake and outcome of routine maternity services in Sierra Leone. | No |
| 32 | Jones et al. 2018 | Liberia | Facility | Qualitative study | Ebola | Pre and post survey | Participatory action research |  | To initiate participatory action research (PAR) in July 2015 to build communication between stakeholder groups, and to identify impacts of the epidemic and shared actions to improve the system. | Yes |
| 33 | Juan et al. 2020 | United States | academic medical centres | Quantitative study | COVID-19 | internet based survey | MFM specialists, or obstetrical service or labor and delivery directors | NA/ 4 sites of MFM consortium | to elucidate the practices put into place to guide patient care after four weeks managing SARS-CoV-2 infections in obstetrical patients | Yes |
| 34 | Karavadra et al. 2020 | United Kingdom | Community | Mixed method study | COVID-19 | 26-item questionnaire (combination of open and closed-ended questions) the questionnaire was advertised through many different online groups and through the BBC news website page to ensure we reached a wide range of participants. | Prenatal and postpartum women | 1552 | 1. to explore pregnant women’s perceptions of COVID-19 and their healthcare experiences. 2. to obtain insight into any barriers to healthcare during this pandemic and any concerns women have about any stage of their pregnancy. | No |
| 35 | Kourouma et al. 2019 | Guinea | public health facilities of the district health of Beyla, Guinea | Quantitative study | Ebola | Secondary(district health office monthly reports) | All women aged 15 to 49 years, children aged from 0 to 11months vaccinated through the extended program of immunization (EPI | NA | to analyze the effects of the 2014 Ebolavirus disease outbreak on antenatal care visits, institutional deliveries and vaccine coverage among children aged 0 to 11months in the health district of Beyla in Guinea | No |
| 36 | Leno et al. 2018 | Guinea | NA | Quantitative study | Ebola | Secondary data from ANC and PMTCT registers | Women | 60 public health centers | to assess the effects of the outbreak on Prevention of Mother-to-Child Transmission of HIV (PMTCT) services in 2014. | No |
| 37 | Li et al. 2020 | China | community | Quantitative study | COVID-19 | Questionnaire | Pregnant women | 2002 | To Investigate the difficulties and needs of pregnant women in the early, middle and late pregnancy stages in the pregnancy and child birth health care services during the epidemic of new coronavirus pneumonia, in order to effectively carry out health education for pregnant women ,strengthen and ,optimize the pregnancy and child birth health management in special periods provide a scientific basis for ensure in the safety of mothers and babies | Yes |
| 38 | Liguoro et al. 2020 | Italy | Facility Based | Quantitative study | COVID-19 | the medical records from the computerized database system of a tertiary level pediatric | Children | 818 | To provide an insight into the change in the use of a pediatric emergency department (ED) during the SARS-CoV-2 pandemic. | No |
| 39 | Luginaah et al. 2016 | Liberia | Community | Mixed method study | Ebola | data were obtained from the women’s files of the 2007 and 2013 Liberia Demographic Health Surveys (LDHS). The LDHS interviews are conducted face-to-face with women aged 15-49 years. | women who have given birth in the five years preceding each survey year. women aged 15-49 years. | 2007 (N = 3524) and 2013 (N = 5127) | this study investigates the timing, access and utilization of maternal care services over time (2007 and 2013) as a background to the post-Ebola epidemic effects on the access to and utilization of care. | No |
| 40 | Lusambili et al. 2020 | Kenya | Semi urban center | Qualitative study | COVID-19 | In-depth interviews | Facility health care workers and community health volunteers ANC and PNC refugee clients | community health volunteers=10 ANC & PNC refugee clients= 15 | To better understand the impact of COVID-19 on access to and use of ANC, delivery and PNC services among refugee women in urban East Leigh. | No |
| 41 | Mahey et al. 2020 | India | Facility Based | Qualitative study | COVID-19 | FGD | Health care workers | 2081 | To determine the impact of roster reorganization on ensuring uninterrupted services while providing necessary relief to healthcare workers (HCW) in the obstetrics department of a tertiary care center amid the COVID‐19 outbreak. | Yes |
| 42 | Marshall et al. 2020 | United States | Facility | Qualitative study | COVID-19 | Structured Questionnaire | Health worker, Administrator | nine statewide weekly focus groups,60 MIECHV staff surveys | describes efforts taken by MIECHV administrators and staff during the pandemic using data collected from 60 MIECHV staff surveys and nine statewide weekly focus groups. | Yes |
| 43 | Masresha et al. 2020 | Multicountry | Community | Quantitative study | COVID-19 | data from the routine immunization programs for the period from January 2018 to June 2020 | children who received BCG vaccine, the first and third doses of Diphtheria-Pertussis- Tetanus containing vaccine (DPT1 and DPT3) and the first and second doses of measles contain in vaccine (MCV1 and MCV2). | Data from 15 countries in African region. | This paper examines the actual routine immunization program performance in selected countries in the African Region by comparing the number of children vaccinated in the early months of the COVID-19 pandemic to the number vaccinated in the months prior to the arrival of COVID-19 in the countries. | No |
| 44 | McDonnell et al. 2020 | Ireland | Facility | Quantitative study | COVID-19 | secondary(ED records) | children under 16 years of age | NA | To assess how the nature of paediatric attendance at the ED altered throughout each of the COVID-19 related public health emergency stages, from the first confirmed case. | No |
| 45 | McQuilkin et al. 2017 | Liberia | community | Quantitative study | Ebola | a structured in-person survey | heads of household | 543 heads of household | The study examined changing patterns of health-care access, barriers to obtaining health care, and outcomes in various patient subgroups including patients seeking care for minor and serious illnesses, malaria treatment, and pediatric, prenatal, and obstetric care. | No |
| 46 | Minckas et al. 2021 | Multicountry | Facility | Quantitative study | COVID-19 | National data on births and proportion of facility deliveries were extracted from the latest Lives Saved Tool (LiST) dataset. | Newborn,  Neonate | 127 counties | To analyze the benefits of kangaroo mother care (KMC) on survival among neonates weighing 2000 g compared with the risk of SARS-CoV-2 acquired from infected mothers/caregivers. | Yes |
| 47 | Nicholson et al. 2020 | Ireland | Each part of Ireland | Quantitative study | COVID-19 | Online questionnaire | parents of children under the age of 16 | 1044 | to understand parents' hesitancy and concerns around accessing healthcare during the pandemic using a cross-sectional survey of parents of children under the age of 16 in Ireland | Yes |
| 48 | Quaglio et al. 2016 | Sierra Leone | Facility | Quantitative study | Ebola | Registers of the two EHCs and contact tracing forms, interview of healthcare workers | NA | 49 case patients | to provide information on understanding of how Ebola impacted maternal and child health services in Sierra Leone. | No |
| 49 | Quaglio et al. 2019 | Sierra Leone | 77 community health facilities and 1 hospital | Quantitative study | Ebola | Data on MCH indicators were prospectively collected from hospital registers. At community level, local district Health Management Information System. | NA | NA | To assess the trends concerning utilization of maternal and child health (MCH) services before, during and after the Ebola outbreak, quantifying the contribution of a re-organized referral system (RS). | No |
| 50 | Ribacke et al. 2016 | Sierra Leone | Facility Based | Quantitative study | Ebola | collected weekly data between January 2014—May 2015 on in hospital deliveries and caesarean sections (C-sections) from all open facilities (public, private for-profit and private non-profit sectors) offering emergency obstetrics in Sierra Leone. | NA | NA | The aim of this study is to assess the potential impact of EVD on nationwide access to obstetric care in Sierra Leone. | No |
| 51 | Rimmer et al. 2020 | United Kingdom | Facility | Mixed method study | COVID-19 | Structured Questionnaire | Junior doctors in obstetrics and gynecology. | 155 | to evaluate the provision of obstetrics and gynecology services in the UK during the acute phase of the COVID‐19 pandemic. | Yes |
| 52 | Salsi et al. 2020 | Italy | Facility | Quantitative study | COVID-19 | Clinical audit of fully anonymized data | Gynecologic patients | 972 | to evaluate the number of women self-referring to the obstetrics and gynecology (OB-GYN) ED triage and the percentage of admission before and during the COVID-19 pandemic | No |
| 53 | Saso et al. 2020 | Multicountry | NA | Mixed method study | COVID-19 | survey using online platform | clinicians and scientists | 48 | To gather a swift snapshot of the global picture and to explore the changes in immunization services experienced by local frontline staffs. | Yes |
| 54 | Semaan et a. 2020 | Multicountry | Facility Based | Qualitative study | COVID-19 | Online survey using questionnaire through professional network and social media in 12 languages. | maternal and newborn health professionals | 714 | The objective of this paper is to synthesize key themes identified in the first round of a global online survey of health professionals working in MNH along four dimensions: preparedness for COVID-19, response to COVID-19, personal experience in the workplace and changes in care provision and processes. | Yes |
| 55 | Siedner et al. 2020 | South Africa | 11 primary healthcare clinics | Quantitative study | COVID-19 | primary | all individuals presented to 11 ambulatory clinic | n=46523 | evaluated whether implementation of lockdown orders in South Africa affected ambulatory clinic visitation in rural Kwa-Zulu Natal (KZN) | Yes |
| 56 | Sigurdsson et al. 2020 | Iceland | Facility | Quantitative study | COVID-19 | medical records database of the PHC | NA | NA | To describe how the primary healthcare (PHC) in Iceland changed its strategy to handle the COVID-19 pandemic. | Yes |
| 57 | Sochas et al. 2017 | Sierra Leone | HMIS | Quantitative study | Ebola | NA | ANC and PNC women | NA | quantifying the extent of the drop in utilization of essential reproductive, maternal and neonatal health services in Sierra Leone during the Ebola outbreak | No |
| 58 | Tadesse 2020 | Ethiopia | Public Hospitals | Quantitative study | COVID-19 | Interviewer administered questionnaire | all pregnant women who attended ANC in the selected health facilities. | n= 389 | to assess the impact of the COVID-19 pandemic on antenatal care utilization among pregnant women attending public facilities in Northeast Ethiopia | No |
| 59 | Williams et al. 2020 | United Kingdom | Facility | Quantitative study | COVID-19 | Secondary data from hospital data base | NA | 273455 unscheduled primary care attendances;  462437 emergency department attendances; 54076 emergency hospital admissions; 413 PICU and 415 deaths | To determine the indirect consequences of the COVID-19 pandemic on paediatric healthcare utilization and severe disease at a national level following lockdown on 23 March 2020. | No |
| 60 | Zhong et al. 2020 | Singapore | public primary care clinics, a hospital paediatric unit, and private pediatrician clinics | Quantitative study | COVID-19 | Secondary(health facility) | Children of immunization age group | NA | to examine vaccination uptake in 3 types of healthcare facilities from January to April 2020 compared to the baseline rates in January to April 2019. | No |
