# Supplementary material for: Effective interventions to ensure MCH (Maternal and Child Health) services during pandemic related health emergencies (Zika, Ebola, and COVID-19): A systematic review
Source: PLoS One. 2022 May 10;17(5):e0268106. doi: 10.1371/journal.pone.0268106 (PMC9089853; doi:10.1371/journal.pone.0268106)
Supplement: S3 File — (DOCX) [file pone.0268106.s004.docx]

Quality Assessment Sheet: Using JBI Critical appraisal checklist for Observational Studies

Prevalence Studies

| Author Year | Q1 | Q2 | Q3 | Q4 | Q5 | Q6 | Q7 | Q8 | Q9 | YES % | Quality* |
| --- | --- | --- | --- | --- | --- | --- | --- | --- | --- | --- | --- |
| Delamou et al. 2017 | Y | U | N | Y | Y | Y | N | Y | U | Y=55% | Moderate |
| Jardine et al. 2020 | Y | U | N | Y | U | U | N | Y | Y | Y=55% | Moderate |
| Nicholson et al. 2020 | Y | Y | U | Y | Y | Y | Y | Y | N | Y=77% | High |
| Garg et al. 2020 | Y | N | U | Y | U | N | N | Y | U | Y=33% | Low |
| Jones et al. 2018 | Y | Y | Y | Y | Y | Y | N | Y | Y | Y=89% | High |
| Li et al. 2020 | Y | U | Y | Y | Y | Y | Y | Y | U | Y=77% | High |
| Bianca et al. 2020 | N | Y | Y | Y | Y | NA | N | Y | Y | Y=77% | High |
| Goyal et al. 2020 | N | N | Y | Y | Y | N | Y | Y | U | Y=55% | Moderate |
| Ribacke et al. 2016 | Y | Y | NA | Y | Y | Y | Y | Y | NA | Y=77% | High |
| Luginaah et al. 2016 | NA | NA | NA | NA | y | y | y | y | Y | Y=77% | High |
| Semaan et a. 2020 | N | N | Y | Y | Y | N | Y | Y | Y | Y=66% | Moderate |

* High Quality > 70%; Moderate Quality 40-70%; Low Quality <40% **Y=Yes; N= No; NA= Not Applicable

Analytical Cross-sectional

| Author Year | Q1 | Q2 | Q3 | Q4 | Q5 | Q6 | Q7 | Q8 | YES% | Quality* |
| --- | --- | --- | --- | --- | --- | --- | --- | --- | --- | --- |
| Gizelis et al. 2017 | Y | Y | N | NA | N | N | Y | Y | Y=57% | Moderate |
| Salsi et al. 2020 | Y | Y | U | U | N | N | N | Y | Y=38% | Low |
| Bechini et al. 2020 | Y | Y | NA | N | N | N | N | Y | 43% | Moderate |
| Minckas et al. 2021 | NA | Y | Y | Y | Y | N | Y | Y | Y=86% | High |
| Rimmer et al. 2020 | Y | Y | Y | N | N | N | Y | Y | Y=63% | Moderate |
| Barden-O'Fallon et al. 2015 | Y | Y | Y | U | Y | N | Y | Y | Y=75% | High |
| Abbas et al. 2020 | Y | NA | Y | Y | Y | N | Y | Y | Y=86% | High |
| Garcia-Huidobro et al. 2020 | Y | Y | Y | N | Y | N | Y | Y | Y=75% | High |
| Indrayani et al. 2020 | Y | Y | Y | N | NA | NA | Y | Y | Y=83% | High |
| Liguoro et al. 2020 | Y | Y | Y | Y | Y | Y | Y | Y | Y=100 | High |
| Homer et al. 2020 | Y | Y | Y | U | N | N | Y | Y | Y=62.5 | Moderate |
| Masresha et al. 2020 | Y | Y | NA | Y | N | N | Y | Y | Y=71.4% | High |
| Jones et al. 2016 | Y | Y | Y | Y | NA | NA | Y | Y | Y=75% | High |
| Siedner et al. 2020 | Y | Y | Y | Y | N | N | Y | Y | Y=75% | High |
| Kourouma et al. 2019 | Y | Y | Y | Y | N | NA | Y | Y | Y=75% | High |
| Tadesse 2020 | Y | Y | Y | Y | N | NA | Y | Y | Y=86% | High |
| Leno et al. 2018 | Y | Y | Y | Y | N | NA | N | Y | Y=71% | High |
| Jensen et al. 2021 | N | N | Y | Y | N | NA | Y | Y | Y=57% | Moderate |
| Sochas et al. 2017 | N | N | Y | Y | N | NA | Y | Y | Y=57% | Moderate |
| Dopfer et al. 2020 | N | Y | Y | Y | N | NA | U | Y | Y=57% | Moderate |
| Bienvenu et al. 2017 | Y | Y | Y | Y | N | NA | Y | Y | Y=86% | High |
| Ashish et al. 2020 | Y | Y | Y | Y | N | NA | Y | Y | Y=86% | High |
| McQuilkin et al. 2017 | NA | Y | Y | Y | N | NA | Y | Y | Y=83% | High |
| Saso et al. 2020 | N | N | Y | Y | N | NA | Y | N | Y=43% | Moderate |
| Chandir et al. 2020 | Y | Y | Y | Y | N | NA | Y | Y | Y=86% | High |
| Quaglio et al. 2019 | NA | Y | Y | Y | N | NA | Y | Y | Y=71% | High |
| Quaglio et al. 2016 | N | Y | N | Y | N | NA | Y | Y | Y=57% | Moderate |
| Chen et al. 2020 | Y | NA | U | Y | N | NA | Y | Y | Y=67% | Moderate |
| Pena et al. 2020 | Y | Y | Y | Y | N | NA | N | N | Y=57% | Moderate |
| Baumann et al. 2020 | Y | Y | Y | Y | N | NA | Y | Y | Y=86% | High |
| Sigurdsson et al. 2020 | N | Y | Y | N | N | NA | Y | Y | Y=57% | Moderate |
| Cella et al. 2020 | NA | Y | N | N | N | NA | U | Y | Y=29% | Low |
| Enyamaa et al. 2020 | Y | Y | Y | Y | N | NA | N | Y | Y=71% | High |
| McDonnell et al. 2020 | Y | Y | Y | Y | N | NA | Y | Y | Y= 86% | High |

* High Quality > 70%; Moderate Quality 40-70%; Low Quality <40% **Y=Yes; N= No; NA= Not Applicable

Qualitative studies

| Study Year | Q1 | Q2 | Q3 | Q4 | Q5 | Q6 | Q7 | Q8 | Q9 | Q10 | YES% | Quality* |
| --- | --- | --- | --- | --- | --- | --- | --- | --- | --- | --- | --- | --- |
| Gizelis et al. 2017 | U | Y | Y | Y | Y | Y | N | Y | Y | Y | Y=70% | High |
| Dynes et al. 2015 | Y | Y | Y | Y | Y | N | N | N | N | Y | Y=60% | Moderate |
| Jones et al. 2018 | Y | Y | Y | Y | Y | N | N | Y | Y | Y | Y=80% | High |
| Marshall et al. 2020 | Y | Y | Y | Y | Y | N | N | Y | Y | Y | Y=80% | High |
| Garcia-Huidobro et al. 2020 | Y | Y | Y | Y | Y | Y | N | Y | Y | Y | Y=90% | High |
| Bianca et al. 2020 | Y | Y | Y | N | Y | N | N | Y | Y | Y | Y=70% | High |
| Fumagalli et al. 2021 | Y | Y | Y | Y | Y | Y | N | Y | Y | Y | Y=90% | High |
| Mahey et al. 2020 | Y | Y | Y | Y | Y | N | N | N | N | Y | Y=60% | Moderate |
| Homer et al. 2020 | Y | Y | Y | Y | Y | N | N | Y | Y | Y | Y=70% | Moderate |
| Semaan et a. 2020 | Y | Y | Y | Y | Y | N | N | Y | Y | Y | Y=80% | High |
| Lusambili et al. 2020 | Y | Y | Y | Y | Y | Y | N | Y | Y | Y | Y=90% | High |
| Karavadra et al. 2020 | Y | Y | Y | Y | Y | N | N | Y | Y | Y | Y=80% | High |
| Hector et al. 2020 | U | Y | Y | Y | Y | N | N | Y | Y | Y | Y=70% | High |
| Belizan et al. 2020 | N | Y | Y | Y | Y | N | N | Y | Y | Y | Y=70% | High |
| Bell et al. 2016 | N | Y | Y | Y | Y | N | N | Y | Y | Y | Y=70% | High |

* High Quality > 70%; Moderate Quality 40-70%; Low Quality <40% **Y=Yes; N= No; NA= Not Applicable

Cohort Studies

| Author Year | Q1 | Q2 | Q3 | Q4 | Q5 | Q6 | Q7 | Q8 | Q9 | Q10 | Q11 | YES% | Quality* |
| --- | --- | --- | --- | --- | --- | --- | --- | --- | --- | --- | --- | --- | --- |
| Hermans et al. 2017 | N | Y | N | N | N | N | y | N | N | N | Y | Y=42% | Low |
| Ferrazzi et al. 2020 | Y | Y | Y | U | N | Y | Y | Y | Y | N | Y | Y=72% | High |
| Zhong et al. 2020 | Y | Y | N | N | N | Y | Y | Y | N | N | Y | Y=75% | High |
| Bornstein et al. 2020 | Y | Y | Y | N | N | Y | Y | N | U | N | Y | Y=67% | High |
| Ahlers-Schmidt et al. 2020 | Y | U | N | N | N | Y | Y | N | N | N | Y | Y=57% | Moderate |
| Williams et al. 2020 | Y | Y | Y | N | N | Y | Y | Y | Y | N | Y | Y=89% | High |
| Delamou et al. 2017 | N | Y | Y | N | N | Y | Y | Y | U | N | Y | Y=55% | Moderate |

* High Quality > 70%; Moderate Quality 40-70%; Low Quality <40% **Y=Yes; N= No; NA= Not Applicable
