# Supplementary material for: Effective interventions to ensure MCH (Maternal and Child Health) services during pandemic related health emergencies (Zika, Ebola, and COVID-19): A systematic review
Source: PLoS One. 2022 May 10;17(5):e0268106. doi: 10.1371/journal.pone.0268106 (PMC9089853; doi:10.1371/journal.pone.0268106)
Supplement: S1 Data — (DOCX) [file pone.0268106.s005.docx]

S1 Data:

Search Strategy

| Concepts | MeSH | Key terms |
| --- | --- | --- |
| Population  Maternal and Child  Health Professionals | "Mothers"[Mesh]  "Pregnant Women"[Mesh]  "Child, Preschool"[Mesh]  "Child"[Mesh]  "Health Personnel"[Mesh] | “Pregnant Woman”  “Postpartum Women*”  Child*  “Health Care Providers*”  “Healthcare Worker*”  “Health Care Professional*”  Doctor*  Nurse*  “community health workers” OR CHW  “Community health volunteers” OR CHV  “Traditional Birth Attendants” OR TBA  ANM OR “Auxiliary Nurse Midwives” |
| Interventions | "Maternal Health Services"[Mesh]  "Child Health Services"[Mesh]  "Maternal-Child Health Services"[Mesh]  "Immunization Programs"[Mesh]  "Prenatal Care"[Mesh]  "Health Care Facilities, Manpower, and Services"[Mesh] | [Maternal Child Health Services](https://www.ncbi.nlm.nih.gov/mesh/2009791)  Antenatal Care  [Perinatal Care](https://www.ncbi.nlm.nih.gov/mesh/68018743)  [Postnatal Care](https://www.ncbi.nlm.nih.gov/mesh/68011181)  [Prenatal Care](https://www.ncbi.nlm.nih.gov/mesh/68011295)  immunization Program  Vaccination*  Obstetric Deliveries  Obstetric Delivery  Institutional delivery |
| Outcome  Quality MCH Care | "Health Care Quality, Access, and Evaluation"[Mesh]  "Quality of HealthCare"[Mesh]  "Facilities and Services Utilization"[Mesh]  "Health Services Accessibility"[Mesh] | Facilities Utilization  Services Utilization  Availability of Health Services  Access to Health Services  Access to Medicines |
| Context | "COVID-19"[Mesh]  "SARS Virus"[Mesh]  "Zika Virus Infection"[Mesh]  "Pandemics"[Mesh]  "Disease Outbreaks"[Mesh]  "Ebolavirus"[Mesh]  "Influenza Pandemic, 1918-1919"[Mesh]  "Epidemics"[Mesh] | “Severe Acute Respiratory Syndrome Virus”  “SARS-Related Coronavirus”  “SARS-CoV”  “SARS Coronavirus”  “ZikV Infection”  “Zika Virus Disease”  “Ebola Virus”  “H1N1 Influenza Pandemic, 1918-1919”  “Infectious Disease Outbreaks”  Epidemic |

**MEDLINE/PubMed**

Concepts:

("Mothers"[Mesh] OR "Pregnant Women"[Mesh] OR "Child, Preschool"[Mesh] OR "Child"[Mesh] OR "Health Personnel"[Mesh] OR “Pregnant Woman” OR “Postpartum Women*” OR Child* OR “Health Care Providers*” OR “Healthcare Worker*” OR “Health Care Professional*” OR Doctor* OR Nurse* OR “community health workers” OR CHW OR ”Community health volunteers” OR CHV OR “Traditional Birth Attendants” OR TBA OR ANM OR “Auxiliary Nurse Midwives”)

AND

("Maternal Health Services"[Mesh] OR "Child Health Services"[Mesh] OR "Maternal-Child Health Services"[Mesh] OR "Immunization Programs"[Mesh] OR "Prenatal Care"[Mesh] OR "Health Care Facilities, Manpower, and Services"[Mesh] OR "Child Health Services" OR "Antenatal Care" OR "Perinatal Care" OR "Postnatal Care" OR "Prenatal Care" OR "immunization Program" OR Vaccination* OR "Obstetric Deliveries" OR "Obstetric Delivery" OR "Institutional delivery") OR ("Health Care Quality, Access, and Evaluation"[Mesh] OR "Quality of Health Care"[Mesh] OR "Facilities and Services Utilization"[Mesh] OR "Health Services Accessibility"[Mesh] OR "Facility Utilization" OR "Service Utilization" OR "Availability of Health Services" OR "Access to Health Service*" OR "Access to Medicines")

AND

("COVID-19"[Mesh] OR "SARS Virus"[Mesh] OR "Zika Virus Infection"[Mesh] OR "Pandemics"[Mesh] OR "Disease Outbreaks"[Mesh] OR "Ebolavirus"[Mesh] OR "Influenza Pandemic, 1918-1919"[Mesh] OR "Epidemics"[Mesh] OR “Severe Acute Respiratory Syndrome Virus” OR “SARS-Related Coronavirus” OR “SARS-CoV” OR “SARS Coronavirus” OR “ZikV Infection” OR “Zika Virus Disease” “Ebola Virus” OR “Infectious Disease Outbreaks” OR Epidemic )

PUBMED SEARCH RESULT:

PubMed Search Result with Filters

CONCEPT: 1 AND 2 AND 3 AND 4 = 526 HITS

(((("Mothers"[Mesh] OR "Pregnant Women"[Mesh] OR "Child, Preschool"[Mesh] OR "Child"[Mesh] OR "Health Personnel"[Mesh] OR "Pregnant Woman"[TIAB] OR "Postpartum Women*"[TIAB] OR Child*[TIAB] OR "Health Care Providers*"[TIAB] OR "Healthcare Worker*"[TIAB] OR "Health Care Professional*"[TIAB] OR Doctor*[TIAB] OR Nurse*[TIAB] OR "community health workers"[TIAB] OR CHW[TIAB] OR "Community health volunteers"[TIAB] OR CHV[TIAB] OR "Traditional Birth Attendants"[TIAB] OR TBA[TIAB] OR ANM[TIAB] OR "Auxiliary Nurse Midwives"[TIAB])) AND (("Maternal Health Services"[Mesh] OR "Child Health Services"[Mesh] OR "Maternal-Child Health Services"[Mesh] OR "Immunization Programs"[Mesh] OR "Prenatal Care"[Mesh] OR "Health Care Facilities, Manpower, and Services"[Mesh] OR "Child Health Services"[TIAB] OR "Antenatal Care"[TIAB] OR "Perinatal Care"[TIAB] OR "Postnatal Care"[TIAB] OR "Prenatal Care"[TIAB] OR "immunization Program"[TIAB] OR Vaccination*[TIAB] OR "Obstetric Deliveries"[TIAB] OR "Obstetric Delivery"[TIAB] OR "Institutional delivery"[TIAB]))) AND (("Health Care Quality, Access, and Evaluation"[Mesh] OR "Quality of Health Care"[Mesh] OR "Facilities and Services Utilization"[Mesh] OR "Health Services Accessibility"[Mesh] OR "Facility Utilization"[TIAB] OR "Service Utilization"[TIAB] OR "Availability of Health Services"[TIAB] OR "Access to Health Service*"[TIAB] OR "Access to Medicines"[TIAB]))) AND (("COVID-19"[Mesh] OR "SARS Virus"[Mesh] OR "Zika Virus Infection"[Mesh] OR "Pandemics"[Mesh] OR "Disease Outbreaks"[Mesh] OR "Ebolavirus"[Mesh] OR "Influenza Pandemic, 1918-1919"[Mesh] OR "Epidemics"[Mesh] OR "Severe Acute Respiratory Syndrome Virus"[TIAB] OR "SARS-Related Coronavirus"[TIAB] OR "SARS-CoV"[TIAB] OR "SARS Coronavirus"[TIAB] OR "ZikV Infection"[TIAB] OR "Zika Virus Disease"[TIAB] OR "Ebola Virus"[TIAB] OR "Infectious Disease Outbreaks"[TIAB] OR Epidemic[TIAB] )) Filters: Clinical Trial, Comparative Study, Multicenter Study, Observational Study, Randomized Controlled Trial

Result: 526 [09/02/2021]

**EBSCO: 11/02/2021**

"Mothers"[MH] OR "Pregnant Women"[MH] OR "Child, Preschool"[MH] OR "Child"[MH] OR "Health Personnel"[MH] OR "Pregnant Woman" OR "Postpartum Women*" OR Child* OR "Health Care Providers*" OR "Healthcare Worker*" OR "Health Care Professional*" OR Doctor* OR Nurse* OR "community health workers" OR CHW OR "Community health volunteers" OR CHV OR "Traditional Birth Attendants" OR TBA OR ANM OR "Auxiliary Nurse Midwives"

AND

"Maternal Health Services"[MH] OR "Child Health Services"[MH] OR "Maternal-Child Health Services"[MH] OR "Immunization Programs"[MH] OR "Prenatal Care"[MH] OR "Health Care Facilities, Manpower, and Services"[MH] OR "Child Health Services" OR "Antenatal Care" OR "Perinatal Care" OR "Postnatal Care" OR "Prenatal Care" OR "immunization Program" OR Vaccination* OR "Obstetric Deliveries" OR "Obstetric Delivery" OR "Institutional delivery"

AND

"Health Care Quality, Access, and Evaluation"[MH] OR "Quality of Health Care"[MH] OR "Facilities and Services Utilization"[MH] OR "Health Services Accessibility"[MH] OR "Facility Utilization" OR "Service Utilization"OR "Availability of Health Services" OR "Access to Health Service*" OR "Access to Medicines"

AND

"COVID-19"[MH] OR "SARS Virus"[MH] OR "Zika Virus Infection"[MH] OR "Pandemics"[MH] OR "Disease Outbreaks"[MH] OR "Ebolavirus"[MH] OR "Influenza Pandemic, 1918-1919"[MH] OR "Epidemics"[MH] OR "Severe Acute Respiratory Syndrome Virus" OR "SARS-Related Coronavirus" OR "SARS-CoV" OR "SARS Coronavirus" OR "ZikV Infection"OR "Zika Virus Disease" OR "Ebola Virus" OR "Infectious Disease Outbreaks" OR Epidemic

1 AND 2 AND 3 AND 4 = 5

**Cochrane**

[mh "Mothers"] OR [mh "Pregnant Women"OR [mh "Child, Preschool"] OR [mh "Child"] OR [mh "Health Personnel"] OR

("Pregnant Woman"):ti,ab OR ("Postpartum Women*"):ti,ab OR (Child*):ti,ab OR ("Health Care Providers*"):ti,ab OR ("Healthcare Worker*"):ti,ab OR ("Health Care Professional*"):ti,ab OR (Doctor*):ti,ab OR (Nurse*):ti,ab OR ("community health workers"):ti,ab OR (CHW):ti,ab OR ("Community health volunteers"):ti,ab OR (CHV):ti,ab OR ("Traditional Birth Attendants"):ti,ab OR (TBA):ti,ab OR (ANM):ti,ab OR ("Auxiliary Nurse Midwives"):ti,ab

AND

"Maternal Health Services"[MH] OR "Child Health Services"[MH] OR "Maternal-Child Health Services"[MH] OR "Immunization Programs"[MH] OR "Prenatal Care"[MH] OR "Health Care Facilities, Manpower, and Services"[MH] OR

("Child Health Services"):ti,ab OR ("Antenatal Care"):ti,ab OR ("Perinatal Care"):ti,ab OR ("Postnatal Care"):ti,ab OR ("Prenatal Care"):ti,ab OR ("immunization Program"):ti,ab OR (Vaccination*):ti,ab OR ("Obstetric Deliveries"):ti,ab OR ("Obstetric Delivery"):ti,ab OR ("Institutional delivery"):ti,ab

AND

"Health Care Quality, Access, and Evaluation"[MH] OR "Quality of Health Care"[MH] OR "Facilities and Services Utilization"[MH] OR "Health Services Accessibility"[MH] OR

("Facility Utilization"):ti,ab OR ("Service Utilization"):ti,ab OR ("Availability of Health Services"):ti,ab OR ("Access to Health Service*"):ti,ab OR ("Access to Medicines"):ti,ab

AND

"COVID-19"[MH] OR "SARS Virus"[MH] OR "Zika Virus Infection"[MH] OR "Pandemics"[MH] OR "Disease Outbreaks"[MH] OR "Ebolavirus"[MH] OR "Influenza Pandemic, 1918-1919"[MH] OR "Epidemics"[MH] OR

("Severe Acute Respiratory Syndrome Virus"):ti,ab OR ("SARS-Related Coronavirus"):ti,ab OR ("SARS-CoV"):ti,ab OR ("SARS Coronavirus"):ti,ab OR ("ZikV Infection"):ti,ab OR ("Zika Virus Disease"):ti,ab OR ("Ebola Virus"):ti,ab OR ("Infectious Disease Outbreaks"):ti,ab OR (Epidemic):ti,ab

Search Name: MCH COVID19 COCHRANE SEARCH

Date Run: 12/02/2021 07:51:04

Comment:

ID Search Hits

#1 MeSH descriptor: [Mothers] explode all trees 1839

#2 MeSH descriptor: [Child] explode all trees 56347

#3 MeSH descriptor: [Pregnant Women] explode all trees 281

#4 MeSH descriptor: [Health Personnel] explode all trees 8974

#5 ("Pregnant Woman"):ti,ab OR ("Postpartum Women*"):ti,ab OR (Child*):ti,ab OR ("Health Care Providers*"):ti,ab OR ("Healthcare Worker*"):ti,ab OR ("Health Care Professional*"):ti,ab OR (Doctor*):ti,ab OR (Nurse*):ti,ab OR ("community health workers"):ti,ab OR (CHW):ti,ab OR ("Community health volunteers"):ti,ab OR (CHV):ti,ab OR ("Traditional Birth Attendants"):ti,ab OR (TBA):ti,ab OR (ANM):ti,ab OR ("Auxiliary Nurse Midwives"):ti,ab 173480

#6 #1 or #2 or #3 or #4 or #5 in Trials with 'Public Health', 'Pregnancy and Childbirth', 'Gynaecology and Fertility', 'Emergency and Critical Care', 'HIV/AIDS', 'Effective Practice and Organisation of Care', 'Neonatal', 'Child Health' in Cochrane Groups 33046

#7 MeSH descriptor: [Maternal Health Services] explode all trees 2269

#8 MeSH descriptor: [Child Health Services] explode all trees 921

#9 MeSH descriptor: [Maternal-Child Health Services] explode all trees 43

#10 MeSH descriptor: [Immunization Programs] explode all trees 215

#11 MeSH descriptor: [Prenatal Care] explode all trees 1508

#12 MeSH descriptor: [Health Care Facilities, Manpower, and Services] explode all trees 106310

#13 ("Child Health Services"):ti,ab OR ("Maternal Health Servivces"):ti,ab OR ("Antenatal Care"):ti,ab OR ("Perinatal Care"):ti,ab OR ("Postnatal Care"):ti,ab OR ("Prenatal Care"):ti,ab OR ("immunization Program"):ti,ab OR (Vaccination*):ti,ab OR ("Obstetric Deliveries"):ti,ab OR ("Obstetric Delivery"):ti,ab OR ("Institutional delivery"):ti,ab 16428

#14 #7 or #8 or #9 or #10 or #11 or #12 or #13 in Trials with 'Public Health', 'Pregnancy and Childbirth', 'Gynaecology and Fertility', 'Emergency and Critical Care', 'HIV/AIDS', 'Effective Practice and Organisation of Care', 'Neonatal', 'Child Health' in Cochrane Groups 16192

#15 MeSH descriptor: [Health Care Quality, Access, and Evaluation] explode all trees 460789

#16 MeSH descriptor: [Quality of Health Care] explode all trees 455104

#17 MeSH descriptor: [Facilities and Services Utilization] explode all trees 31

#18 MeSH descriptor: [Health Services Accessibility] explode all trees 962

#19 ("Facility Utilization"):ti,ab OR ("Service Utilization"):ti,ab OR ("Availability of Health Services"):ti,ab OR ("Access to Health Service*"):ti,ab OR ("Access to Medicines"):ti,ab 932

#20 #15 or #16 or #17 or #18 or #19 in Trials with 'Public Health', 'Pregnancy and Childbirth', 'Gynaecology and Fertility', 'Emergency and Critical Care', 'HIV/AIDS', 'Effective Practice and Organisation of Care', 'Neonatal', 'Child Health' in Cochrane Groups 43932

#21 MeSH descriptor: [COVID-19] explode all trees 189

#22 MeSH descriptor: [SARS Virus] explode all trees 9

#23 MeSH descriptor: [Zika Virus Infection] explode all trees 10

#24 MeSH descriptor: [Pandemics] explode all trees 207

#25 MeSH descriptor: [Disease Outbreaks] explode all trees 437

#26 MeSH descriptor: [Ebolavirus] explode all trees 28

#27 MeSH descriptor: [Hemorrhagic Fever, Ebola] explode all trees 58

#28 MeSH descriptor: [Epidemics] explode all trees 233

#29 ("Severe Acute Respiratory Syndrome Virus"):ti,ab OR ("SARS-Related Coronavirus"):ti,ab OR ("SARS-CoV"):ti,ab OR ("SARS Coronavirus"):ti,ab OR ("ZikV Infection"):ti,ab OR ("Zika Virus Disease"):ti,ab OR ("Ebola Virus"):ti,ab OR ("Infectious Disease Outbreaks"):ti,ab OR (Epidemic):ti,ab 2976

#30 #21 or #22 or #23 or #24 or #25 or #26 or #27 or #28 or #29 in Trials with 'Public Health', 'Pregnancy and Childbirth', 'Gynaecology and Fertility', 'Emergency and Critical Care', 'HIV/AIDS', 'Effective Practice and Organisation of Care', 'Neonatal', 'Child Health' in Cochrane Groups 183

#31 #6 and #14 and #20 and #30 38

LINK FOR RESULT: [Search Manager | Cochrane Library](https://www.cochranelibrary.com/web/cochrane/advanced-search/search-manager)

**ScienceDirect**: 688 Hits [17/02/2021]

(Maternal OR Mother OR "Pregnant woman" OR Neonates OR Infants OR Child) AND (Maternal and Child health Service) AND (COVID19 OR Pandemic)

Search strategy for ScienceDirect; 688 results

**Epistemonikos**: 17 [17/02/2021]

(title:((title:("Pregnant Woman") OR abstract:("Pregnant Woman")) OR (title:(Postpartum Women) OR abstract:(Postpartum Women)) OR (title:(Children) OR abstract:(Children)) OR (title:(child) OR abstract:(child))) OR abstract:((title:("Pregnant Woman") OR abstract:("Pregnant Woman")) OR (title:(Postpartum Women) OR abstract:(Postpartum Women)) OR (title:(Children) OR abstract:(Children)) OR (title:(child) OR abstract:(child)))) AND (title:((title:(Maternal Child Health Services) OR abstract:(Maternal Child Health Services)) OR (title:(Antenatal Care) OR abstract:(Antenatal Care)) OR (title:(Perinatal Care) OR abstract:(Perinatal Care)) OR (title:(Postnatal Care) OR abstract:(Postnatal Care)) OR (title:(Prenatal Care) OR abstract:(Prenatal Care)) OR (title:(immunization Program) OR abstract:(immunization Program)) OR (title:(Vaccination) OR abstract:(Vaccination)) OR (title:(Obstetric Deliveries) OR abstract:(Obstetric Deliveries)) OR (title:(Institutional delivery) OR abstract:(Institutional delivery))) OR abstract:((title:(Maternal Child Health Services) OR abstract:(Maternal Child Health Services)) OR (title:(Antenatal Care) OR abstract:(Antenatal Care)) OR (title:(Perinatal Care) OR abstract:(Perinatal Care)) OR (title:(Postnatal Care) OR abstract:(Postnatal Care)) OR (title:(Prenatal Care) OR abstract:(Prenatal Care)) OR (title:(immunization Program) OR abstract:(immunization Program)) OR (title:(Vaccination) OR abstract:(Vaccination)) OR (title:(Obstetric Deliveries) OR abstract:(Obstetric Deliveries)) OR (title:(Institutional delivery) OR abstract:(Institutional delivery)))) AND (title:((title:(Facilities Utilization) OR abstract:(Facilities Utilization)) OR (title:(Services Utilization) OR abstract:(Services Utilization)) OR (title:(Availability of Health Services) OR abstract:(Availability of Health Services)) OR (title:(Access to Health Services) OR abstract:(Access to Health Services)) OR (title:(Access to Medicines) OR abstract:(Access to Medicines))) OR abstract:((title:(Facilities Utilization) OR abstract:(Facilities Utilization)) OR (title:(Services Utilization) OR abstract:(Services Utilization)) OR (title:(Availability of Health Services) OR abstract:(Availability of Health Services)) OR (title:(Access to Health Services) OR abstract:(Access to Health Services)) OR (title:(Access to Medicines) OR abstract:(Access to Medicines)))) AND (title:((title:("Severe Acute Respiratory Syndrome Virus") OR abstract:("Severe Acute Respiratory Syndrome Virus")) OR (title:(SARS-Related Coronavirus") OR abstract:(SARS-Related Coronavirus")) OR (title:("SARS-CoV") OR abstract:("SARS-CoV")) OR (title:(SARS Coronavirus) OR abstract:(SARS Coronavirus)) OR (title:(ZikV Infection) OR abstract:(ZikV Infection)) OR (title:(Zika Virus Disease) OR abstract:(Zika Virus Disease)) OR (title:(Ebola Virus) OR abstract:(Ebola Virus)) OR (title:(H1N1 Influenza Pandemic) OR abstract:(H1N1 Influenza Pandemic)) OR (title:(Infectious Disease Outbreaks) OR abstract:(Infectious Disease Outbreaks)) OR (title:(Epidemic) OR abstract:(Epidemic)) OR (title:(Pandemic) OR abstract:(Pandemic))) OR abstract:((title:("Severe Acute Respiratory Syndrome Virus") OR abstract:("Severe Acute Respiratory Syndrome Virus")) OR (title:(SARS-Related Coronavirus") OR abstract:(SARS-Related Coronavirus")) OR (title:("SARS-CoV") OR abstract:("SARS-CoV")) OR (title:(SARS Coronavirus) OR abstract:(SARS Coronavirus)) OR (title:(ZikV Infection) OR abstract:(ZikV Infection)) OR (title:(Zika Virus Disease) OR abstract:(Zika Virus Disease)) OR (title:(Ebola Virus) OR abstract:(Ebola Virus)) OR (title:(H1N1 Influenza Pandemic) OR abstract:(H1N1 Influenza Pandemic)) OR (title:(Infectious Disease Outbreaks) OR abstract:(Infectious Disease Outbreaks)) OR (title:(Epidemic) OR abstract:(Epidemic)) OR (title:(Pandemic) OR abstract:(Pandemic))))

**EMBASE**

'mother'/exp OR 'pregnant woman'/exp OR 'preschool child'/exp OR 'health care personnel'/exp OR 'health auxiliary'/exp OR 'traditional birth attendant'/exp OR ("Pregnant Woman"):ti,ab OR ("Postpartum Women*"):ti,ab OR (Child*):ti,ab OR ("Health Care Providers*"):ti,ab OR ("Healthcare Worker*"):ti,ab OR ("Health Care Professional*"):ti,ab OR (Doctor*):ti,ab OR (Nurse*):ti,ab OR ("community health workers"):ti,ab OR ("Auxiliary Nurse Midwives"):ti,ab OR ("Traditional Birth Attendants"):ti,ab OR TBA OR ANM OR ("Auxiliary Nurse Midwives"):ti,ab

AND

'maternal health service'/exp OR 'maternal child health care'/exp OR 'child health care'/exp OR 'health service'/exp OR 'prenatal care'/exp OR 'health care facilities and services'/exp OR 'perinatal care'/exp OR 'postnatal care'/exp OR 'immunization program'/exp OR 'institutional delivery'/exp OR ("Maternal Health Services"):ti,ab OR ("Child Health Services"):ti,ab OR ("Maternal-Child Health Services"):ti,ab OR ("Immunization Programs"):ti,ab OR ("Prenatal Care"):ti,ab OR ("Health Care Facilities, Manpower, and Services"):ti,ab OR ("Child Health Services"):ti,ab OR ("Antenatal Care"):ti,ab OR ("Perinatal Care"):ti,ab OR ("Postnatal Care"):ti,ab OR ("Prenatal Care"):ti,ab OR ("immunization Program"):ti,ab OR (Vaccination*):ti,ab OR ("Obstetric Deliveries"):ti,ab OR ("Obstetric Delivery"):ti,ab OR ("Institutional delivery"):ti,ab

AND

'health care quality'/exp OR 'facilities and services utilization'/exp OR 'health care access'/exp OR 'health care utilization'/exp OR ("Health Care Quality, Access, and Evaluation"):ti,ab OR ("Quality of Health Care"):ti,ab OR ("Facilities and Services Utilization"):ti,ab OR ("Health Services Accessibility"):ti,ab OR ("Facility Utilization"):ti,ab OR ("Service Utilization"):ti,ab OR ("Availability of Health Services"):ti,ab OR ("Access to Health Service*"):ti,ab OR ("Access to Medicines"):ti,ab

AND

'coronavirus disease 2019'/exp OR 'SARS coronavirus'/exp OR 'Zika fever'/exp OR 'pandemic'/exp OR 'epidemic'/exp OR 'Ebolavirus'/exp OR 'Spanish influenza'/exp OR 'Zika fever'/exp OR ("COVID-19"):ti,ab OR ("SARS Virus"):ti,ab OR ("Zika Virus Infection"):ti,ab OR ("Pandemics"):ti,ab OR ("Disease Outbreaks"):ti,ab OR ("Ebolavirus"):ti,ab OR ("Influenza Pandemic, 1918-1919"):ti,ab OR ("Epidemics"):ti,ab OR ("Severe Acute Respiratory Syndrome Virus"):ti,ab OR ("SARS-Related Coronavirus"):ti,ab OR ("SARS-CoV"):ti,ab OR ("SARS Coronavirus"):ti,ab OR ("ZikV Infection"):ti,ab OR ("Zika Virus Disease"):ti,ab OR ("Ebola Virus"):ti,ab OR ("Infectious Disease Outbreaks"):ti,ab OR (Epidemic):ti,ab

Embase Session Results

No.Query Results

4,345

#6

#5 AND ('case control study'/de OR 'clinical article'/de OR 'clinical trial'/de OR 'cohort analysis'/de OR 'comparative effectiveness'/de OR 'comparative study'/de OR 'controlled clinical trial'/de OR 'controlled study'/de OR 'cross sectional study'/de OR 'evidence based medicine'/de OR 'evidence based practice'/de OR 'intervention study'/de OR 'interview'/de OR 'longitudinal study'/de OR 'major clinical study'/de OR 'multicenter study'/de OR 'observational study'/de OR 'pilot study'/de OR 'prospective study'/de OR 'qualitative research'/de OR 'questionnaire'/de OR 'randomized controlled trial'/de OR 'retrospective study'/de OR 'semi structured interview'/de)

9,792

#5

#1 AND #2 AND #3 AND #4

248,135

#4

('coronavirus disease 2019'/exp OR 'coronavirus disease 2019' OR 'sars coronavirus'/exp OR 'sars coronavirus' OR 'pandemic'/exp OR 'pandemic' OR 'epidemic'/exp OR 'epidemic' OR 'ebolavirus'/exp OR 'ebolavirus' OR 'spanish influenza'/exp OR 'spanish influenza' OR 'zika fever'/exp OR 'zika fever' OR 'covid-19':ti,ab OR 'sars virus':ti,ab OR 'zika virus infection':ti,ab OR 'pandemics':ti,ab OR 'disease outbreaks':ti,ab OR 'ebolavirus':ti,ab OR 'influenza pandemic, 1918-1919':ti,ab OR 'epidemics':ti,ab OR 'severe acute respiratory syndrome virus':ti,ab OR 'sars-related coronavirus':ti,ab OR 'sars-cov':ti,ab OR 'sars coronavirus':ti,ab OR 'zikv infection':ti,ab OR 'zika virus disease':ti,ab OR 'ebola virus':ti,ab OR 'infectious disease outbreaks':ti,ab OR epidemic:ti,ab) AND [embase]/lim

2,764,331

#3

('health care quality'/exp OR 'health care quality' OR 'facilities and services utilization'/exp OR 'facilities and services utilization' OR 'health care access'/exp OR 'health care access' OR 'health care utilization'/exp OR 'health care utilization' OR 'health care quality, access, and evaluation':ti,ab OR 'quality of health care':ti,ab OR 'facilities and services utilization':ti,ab OR 'health services accessibility':ti,ab OR 'facility utilization':ti,ab OR 'service utilization':ti,ab OR 'availability of health services':ti,ab OR 'access to health service*':ti,ab OR 'access to medicines':ti,ab) AND [embase]/lim

5,499,107

#2

('maternal health service'/exp OR 'maternal health service' OR 'maternal child health care'/exp OR 'maternal child health care' OR 'child health care'/exp OR 'child health care' OR 'health service'/exp OR 'health service' OR 'prenatal care'/exp OR 'prenatal care' OR 'health care facilities and services'/exp OR 'health care facilities and services' OR 'perinatal care'/exp OR 'perinatal care' OR 'postnatal care'/exp OR 'postnatal care' OR 'immunization program'/exp OR 'immunization program' OR 'institutional delivery'/exp OR 'institutional delivery' OR 'maternal health services':ti,ab OR 'maternal-child health services':ti,ab OR 'immunization programs':ti,ab OR 'health care facilities, manpower, and services':ti,ab OR 'child health services':ti,ab OR 'antenatal care':ti,ab OR 'perinatal care':ti,ab OR 'postnatal care':ti,ab OR 'prenatal care':ti,ab OR 'immunization program':ti,ab OR vaccination*:ti,ab OR 'obstetric deliveries':ti,ab OR 'obstetric delivery':ti,ab OR 'institutional delivery':ti,ab) AND [embase]/lim

2,997,327

#1

('mother'/exp OR 'mother' OR 'pregnant woman'/exp OR 'pregnant woman' OR 'preschool child'/exp OR 'preschool child' OR 'health care personnel'/exp OR 'health care personnel' OR 'health auxiliary'/exp OR 'health auxiliary' OR 'traditional birth attendant'/exp OR 'traditional birth attendant' OR 'pregnant woman':ti,ab OR 'postpartum women*':ti,ab OR child*:ti,ab OR 'health care providers*':ti,ab OR 'healthcare worker*':ti,ab OR 'health care professional*':ti,ab OR doctor*:ti,ab OR nurse*:ti,ab OR 'community health workers':ti,ab OR 'traditional birth attendants':ti,ab OR 'tba'/exp OR tba OR anm OR 'auxiliary nurse midwives':ti,ab) AND [embase]/lim

CONCEPT:

"Mothers" OR "Pregnant Women" OR "Child, Preschool" OR "Child" OR "Health Personnel" OR "Pregnant Woman" OR "Postpartum Women*" OR Child* OR "Health Care Providers*" OR "Healthcare Worker*" OR "Health Care Professional*" OR Doctor* OR Nurse* OR "community health workers" OR CHW OR "Auxiliary Nurse Midwives" OR CHV OR "Traditional Birth Attendants" OR TBA OR ANM OR "Auxiliary Nurse Midwives"

AND

"Maternal Health Services" OR "Child Health Services" OR "Maternal-Child Health Services" OR "Immunization Programs" OR "Prenatal Care" OR "Health Care Facilities, Manpower, and Services" OR "Child Health Services" OR "Antenatal Care" OR "Perinatal Care" OR "Postnatal Care" OR "Prenatal Care" OR "immunization Program" OR Vaccination* OR "Obstetric Deliveries" OR "Obstetric Delivery" OR "Institutional delivery"

AND

"Health Care Quality, Access, and Evaluation" OR "Quality of Health Care" OR "Facilities and Services Utilization" OR "Health Services Accessibility" OR "Facility Utilization" OR "Service Utilization"OR "Availability of Health Services" OR "Access to Health Service*" OR "Access to Medicines"

AND

"COVID-19" OR "SARS Virus" OR "Zika Virus Infection" OR "Pandemics" OR "Disease Outbreaks" OR "Ebolavirus" OR "Influenza Pandemic, 1918-1919" OR "Epidemics" OR "Severe Acute Respiratory Syndrome Virus" OR "SARS-Related Coronavirus" OR "SARS-CoV" OR "SARS Coronavirus" OR "ZikV Infection"OR "Zika Virus Disease" OR "Ebola Virus" OR "Infectious Disease Outbreaks" OR Epidemic

**Google Scholar:**

Search strategy:

("Mothers" OR "Pregnant Women" OR “Postpartum Woman” OR "Child health OR "Child") AND ("Maternal Health Services" OR "Child Health Services" OR "Prenatal Care" OR "Antenatal Care" OR "Perinatal Care" OR "Postnatal Care" OR "Prenatal Care") AND ("Facilities and Services Utilization" OR "Facility Utilization" OR "Service Utilization" OR "Availability of Health Services" OR "Access to Health Service") AND ("COVID-19" OR "Pandemics" OR "Disease Outbreaks" OR "Epidemics" OR “Infectious Disease Outbreaks” OR Epidemic): 17,200

(Maternal health OR child health OR pregnant women) AND (Maternal health services OR prenatal care OR antenatal care OR postnatal care) AND (Facility utilization OR facility services) AND (COVID-19 OR Disease outbreaks OR epidemic): 38,600

After Title Screened: 46
